# Unraveling the evolution and coevolution of small regulatory RNAs and coding genes in *Listeria*

**DOI:** 10.1186/s12864-017-4242-0

**Published:** 2017-11-16

**Authors:** Franck Cerutti, Ludovic Mallet, Anaïs Painset, Claire Hoede, Annick Moisan, Christophe Bécavin, Mélodie Duval, Olivier Dussurget, Pascale Cossart, Christine Gaspin, Hélène Chiapello

**Affiliations:** 1 Université de Toulouse, INRA, UR 875 Unité Mathématiques et Informatique Appliquées de Toulouse, Auzeville, 31326 Castanet-Tolosan, France; 2 Département de Biologie Cellulaire et Infection, Institut Pasteur, Unité des Interactions Bactéries-Cellules, F-75015 Paris, France; 3 INSERM, U604,F-75015 Paris, France; 4 INRA, USC2020, F-75015 Paris, France; 5 Institut Pasteur – Bioinformatics and Biostatistics Hub – C3BI, USR 3756 IP CNRS, Paris, France; 6 Université Paris Diderot, Sorbonne Paris Cité, F-75013 Paris, France; 7 Present address: Public Health England, 61 Colindale Avenue, London, NW9 5EQ England

**Keywords:** *Listeria*, sRNA, Phylogenomics, Coevolution network, Regulation, Cell wall, Pathogenicity, Internalin

## Abstract

**Background:**

Small regulatory RNAs (sRNAs) are widely found in bacteria and play key roles in many important physiological and adaptation processes. Studying their evolution and screening for events of coevolution with other genomic features is a powerful way to better understand their origin and assess a common functional or adaptive relationship between them. However, evolution and coevolution of sRNAs with coding genes have been sparsely investigated in bacterial pathogens.

**Results:**

We designed a robust and generic phylogenomics approach that detects correlated evolution between sRNAs and protein-coding genes using their observed and inferred patterns of presence-absence in a set of annotated genomes. We applied this approach on 79 complete genomes of the *Listeria* genus and identified fifty-two accessory sRNAs, of which most were present in the *Listeria* common ancestor and lost during *Listeria* evolution. We detected significant coevolution between 23 sRNA and 52 coding genes and inferred the *Listeri*a sRNA-coding genes coevolution network. We characterized a main hub of 12 sRNAs that coevolved with genes encoding cell wall proteins and virulence factors. Among them, an sRNA specific to *L. monocytogenes* species, *rli133,* coevolved with genes involved either in pathogenicity or in interaction with host cells, possibly acting as a direct negative post-transcriptional regulation.

**Conclusions:**

Our approach allowed the identification of candidate sRNAs potentially involved in pathogenicity and host interaction, consistent with recent findings on known pathogenicity actors. We highlight four sRNAs coevolving with seven internalin genes, some of which being important virulence factors in *Listeria*.

**Electronic supplementary material:**

The online version of this article (10.1186/s12864-017-4242-0) contains supplementary material, which is available to authorized users.

## Background

Small regulatory RNAs are widespread in all kingdoms of life and are recognized as key negative or positive regulators of gene expression [1–3]. They are involved in a wide panel of physiological processes and adaptive responses in bacteria including stress responses, quorum sensing, toxin-antitoxin systems or pathogenicity [4–6]. They generally act post-transcriptionally in *cis* (antisense) or *trans* by base pairing with their target messenger RNA (mRNA) but can also bind specific proteins and modify their activity, as illustrated by CsrB and 6S sRNA [1]. The most extensively studied class of sRNA includes *trans*-encoded sRNAs which regulate their target mRNA by forming short and imperfect duplexes. In silico identification of these duplexes remains a major challenge due to a prohibitively high level of false positive candidates [7–9]. Nevertheless, an improvement in target prediction was shown [10, 11] by focusing on site-specific regions such as the ribosome binding site (RBS), the accessibility of unstructured seed regions in both the sRNA and target mRNA, and the use of comparative genomics of interaction candidates. Altogether, these features argue for a better understanding of sRNA history during bacterial evolution and shed light on how regulatory networks involving *trans*-acting-sRNA and target mRNA have emerged and evolved. Unfortunately, little is known about sRNA evolution, sRNA expression control and sRNA-mRNA coevolution within bacteria, and very few studies have been carried out on these topics so far. This can be explained by the lack of sRNA annotation in available genome resources as well as by the low number of well-characterized regulatory sRNAs and the rapid evolution of regulatory sRNAs in bacteria [1].

In the last decade, high throughput sequencing and transcriptome-wide approaches led to a continuous accumulation of complete genomic data in public databases and contributed to the discovery of hundreds of putative and confirmed new sRNAs in many bacteria such as *Escherichia coli* [12], *Salmonella* [13], *Bacillus subtilis* [14, 15] and *Listeria* [5, 6, 16–27], giving rise to large-scale comparative analyses and sRNA evolutionary studies. Existing studies on that topic focused on Gram-negative species, including *Escherichia coli* and related genomes [8, 28–30]. Phyletic analysis of *E.coli* sRNAs [29] led to the first insights into the distribution of sRNAs in gamma-proteobacteria, greatly improving our understanding of the origin of sRNA-mediated regulation and the underlying mechanisms at the source of sRNA acquisition. To our knowledge, such a global evolutionary study has never been performed in Gram-positive bacteria.

*Listeria* are Gram-positive bacteria that are widespread in the environment and encompass 17 species, two of which are pathogenic: *Listeria monocytogenes*, the human foodborne agent responsible for listeriosis, and *Listeria ivanovii*, an animal pathogen [31]. *L. monocytogenes* has become a model for the study of host-pathogen interactions due to its unique ability to cross host barriers, escape from immune defenses, invade cells and manipulate cellular machineries [32–34]. The comparative analysis of the complete genome sequence of *L. monocytogenes* and the non pathogenic species *L. innocua* in 2001 was the first study to shed light on *Listeria* virulence and its evolution [35]. Following this pioneer work, *Listeria* genomic data grew exponentially and more than 80 complete genomes have been sequenced [36]. Small non-coding RNAs were also extensively studied in *L. monocytogenes* [5, 6, 16–27]. Indeed, 304 non-coding RNAs elements were reported in *L. monocytogenes* EGD-e including 154 sRNAs, 104 antisense and 46 *cis*-encoded [16, 17, 37]. Among these sRNAs, several were shown to be upregulated in bacteria growing in murine intestinal lumen and in human blood, suggesting that they may play a role in adaptation of the bacteria to the niches occupied during infection [1, 5, 21].

Comparative analyses of *Listeria* sRNAs by Kuenne et al. [38] revealed the organization of CRISPR arrays and *cas* genes in 38 complete *L. monocytogenes* genomes. Becavin et al. compared three *L. monocytogenes* species and observed a high conservation of sRNAs compared to protein-coding genes [37]. A comparative transcriptomics approach was also used to compare the expression of non-coding RNAs in *L. monocytogenes* and *L. innocua* species, which revealed conservation across most transcripts, but significant divergence between the species in a subset of non-coding sRNAs [22].

In this article, we present a robust phylogenomics approach that extends and improves existing strategies dedicated to the study of sRNA evolutionary dynamics. We use it to provide the first evolutionary dynamics study of 79 complete genomes of the *Listeria* genus with regards to protein-coding genes, and a selected set of 112 sRNA loci experimentally identified in the pathogenic strain *L.monocytogenes* EGD-e. This dataset includes intergenic trans-encoded sRNAs assumed to target independently expressed and distant mRNAs. We built the core and accessory sRNA and coding genes sets and deduced the ancestral presence-absence states for all *Listeria* genes. Using these patterns, we identified a subset of 23 sRNAs that significantly coevolved with 5′ untranslated regions of coding genes (5’UTRs) and coding DNA sequence (CDS) regions of 52 *Listeria* coding genes. We reconstructed the coevolution network between sRNAs and coding genes and revealed a hub of 12 sRNAs coevolving with genes encoding cell wall proteins and virulence factors. Among them, we focused on *rli133*, an sRNA specific of *L. monocytogenes* species that coevolved with 12 coding genes, six of which exhibited a documented function linked to either virulence or interaction with the host cell, possibly acting as a negative post-transcriptional regulator.

## Results

### A robust screening strategy for sRNA and coding genes coevolution

**Fig. 1:**
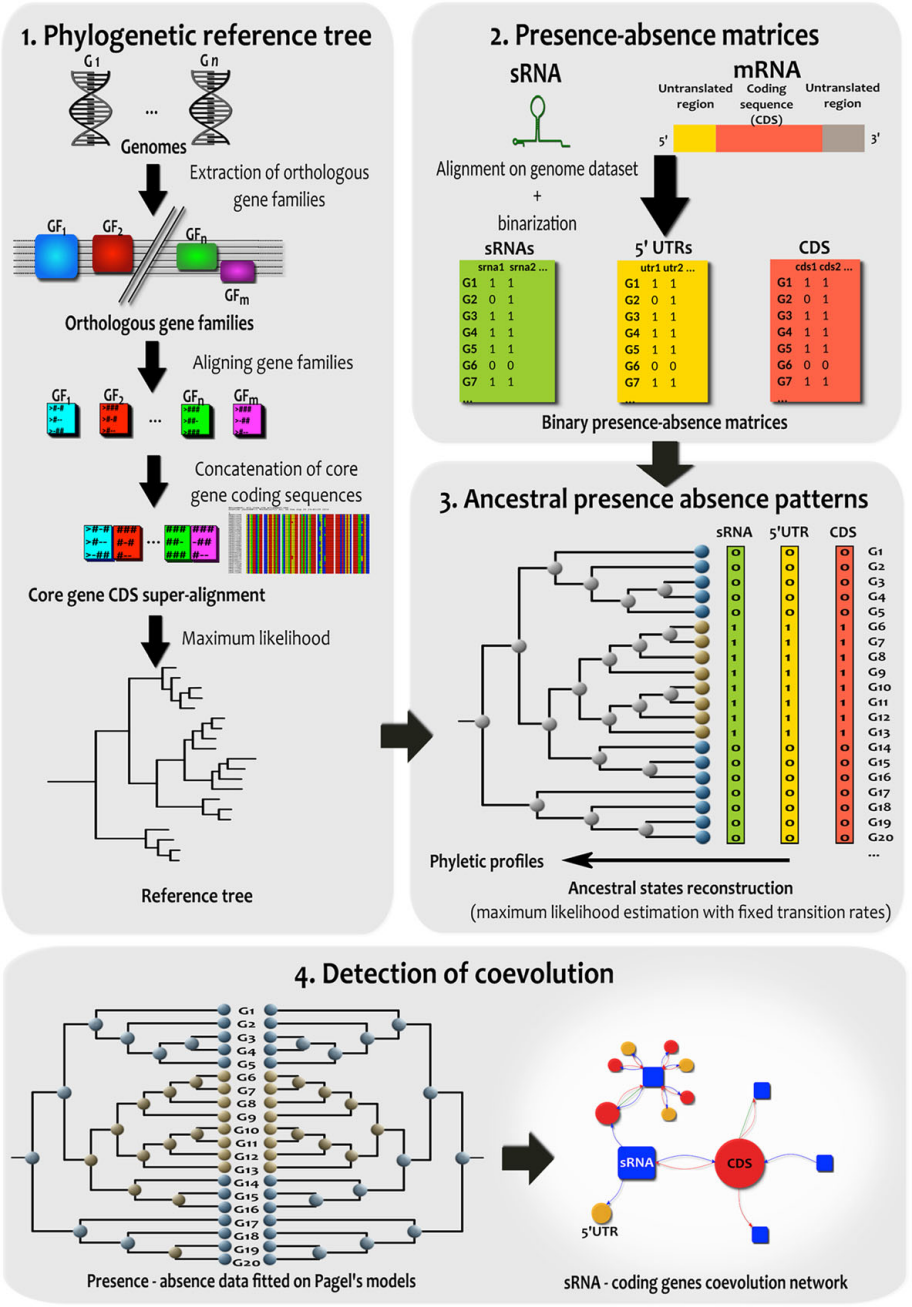
Strategy and workflow. The strategy consists in 4 steps: (1) Construction of a phylogenetic reference tree computed from a super-alignment of syntenic core genes and a Maximum Likelihood approach (2) Presence-absence matrices computation using alignments of sRNAs, 5’UTRs and CDS (3) Ancestral presence-absence pattern reconstruction for sRNAs, 5’UTRs and CDS based on Markov Model and a Maximum Likelihood approach (4) Detection of coevolution events between sRNAs and 5’UTRs or CDS using both observed and ancestral patterns and construction of the sRNA-coding genes coevolution network

We designed an original approach to build a reference phylogenetic tree to infer observed and ancestral evolution patterns and to identify coevolution relationships between pairs of sRNAs and coding-genes. The four main steps of this approach are presented in Fig. 1 and a full description of each step of the workflow is provided in the Methods section. Briefly, the approach starts from a set of annotated genomes and a list of sRNAs to proceed through four main steps: (1) the construction of a reference phylogenetic tree based on orthologous genes; (2) the construction of the presence-absence matrix for sRNAs, 5’UTRs and CDS parts allowing across all the genomes to define core and accessory sets for all elements; (3) the inference of ancestral presence-absence patterns for all variable sRNAs, 5’UTRs and CDS; and (4) the detection of coevolution events between regulatory sRNA and coding genes and construction of a coevolution network using both the observed and the reconstructed ancestral presence-absence patterns. The detection of correlated evolution events relies on a phylogenetic-statistical method based on continuous-time Markov modeling of trait evolution developed by M. Pagel [39]. It compares the statistical likelihood of the observed data (in this case, sRNAs, 5’UTRs and CDS presence/absence patterns) under two alternative scenarii: one in which the two features are allowed to evolve independently on the phylogeny, and another where they coevolve together.

This strategy was applied on 112 *L. monocytogenes* EGD-e putative trans sRNAs, all screened on 79 *Listeria* genomes (see Additional file 1: Table S1) obtained from the Listeriomics database [36]. To deal with the remaining paralogs in the dataset, sRNAs exhibiting overlapping positions on the EGD-e reference genome were merged in 15 sRNA loci (see Additional file 2: Table S2 and the Methods section for details).

**Fig. 2:**
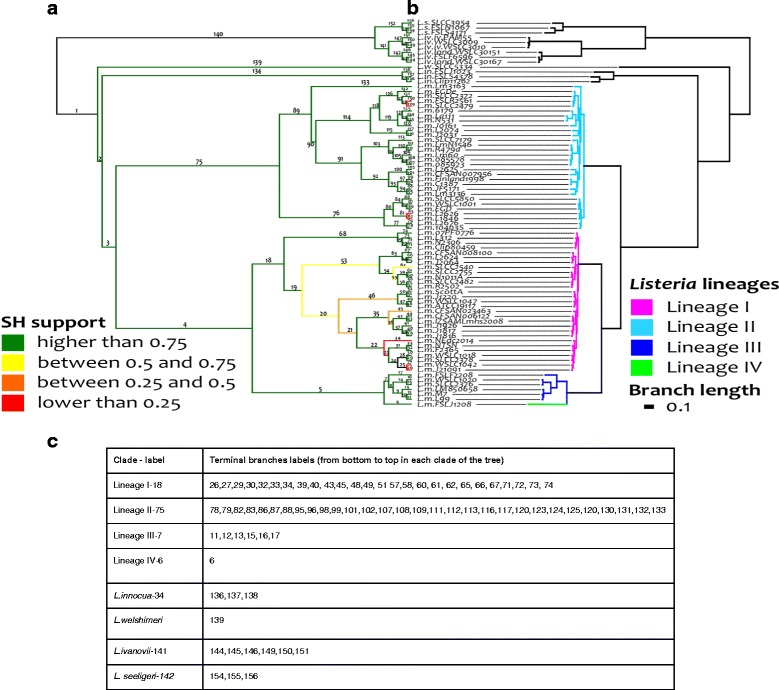
*Listeria* reference tree. The left part (**a**) presents the tree as a cladogram to visualize Shimodaira–Hasegawa (SH) supports for all branches. Best SH support branches (SH support values >0.75) are indicated in green. Branches with a support value between 0.5 and 0.75 are indicated in yellow and those with a support value between 0.25 and 0.5 are indicated in orange. Low SH support branches are indicated in red (SH support values <0.25). The right part (**b**) represents the tree with estimated branch lengths. The four highlighted clades correspond to the four known Listeria lineages. Branch labels are used in Additional file 4: Table S4. Terminal branch labels of each main clade of the tree are listed in a separated table below (**c**)

The *Listeria* phylogenetic reference tree obtained from the 1399 syntenic core coding genes of *Listeria* was robust and consistent with previous studies [40] (see Fig. 2). The four major phylogenetic lineages of *L. monocytogenes* were clearly separated with good Shimodaira Hasegawa (SH) supports (Fig. 2b) [40]. We however observed a few branches of lineage I with lower SH support that correspond to highly conserved genomes, resulting in short branch lengths and a weaker phylogenetic signal. This reference tree was subsequently used to compare sRNAs and coding gene content of *Listeria* genomes.

### sRNA content of *Listeria* genomes

On the 112 *L. monocytogenes* EGD-e sRNAs, 52 (46%) were found to be variable in *Listeria* genomes, i.e., absent in at least one *Listeria* genome, and 60 (54%) were found to be present in all *Listeria* genomes (Additional file 3: Table S3) the later constitute the core *Listeria*-sRNAs. Six of these core sRNAs (rli102, rli119, rli120, rli19-ssrA, rli69 and the rli2-LhrC-2_rli4-LhrC-4_rli7-LhrC-5_rli3-LhrC-3_rli1-LhrC-1 sRNA locus) were kept in the core set despite that their presence could not be confirmed in one or two genomes due to unsequenced regions. Among the core sRNAs, 79% of their occurrences were located in syntenic regions (meaning that both 5′ and 3′ adjacent genes were also found conserved). We then focused on the 52 variable sRNA loci to decipher their evolutionary history in *Listeria* genomes.

**Fig. 3:**
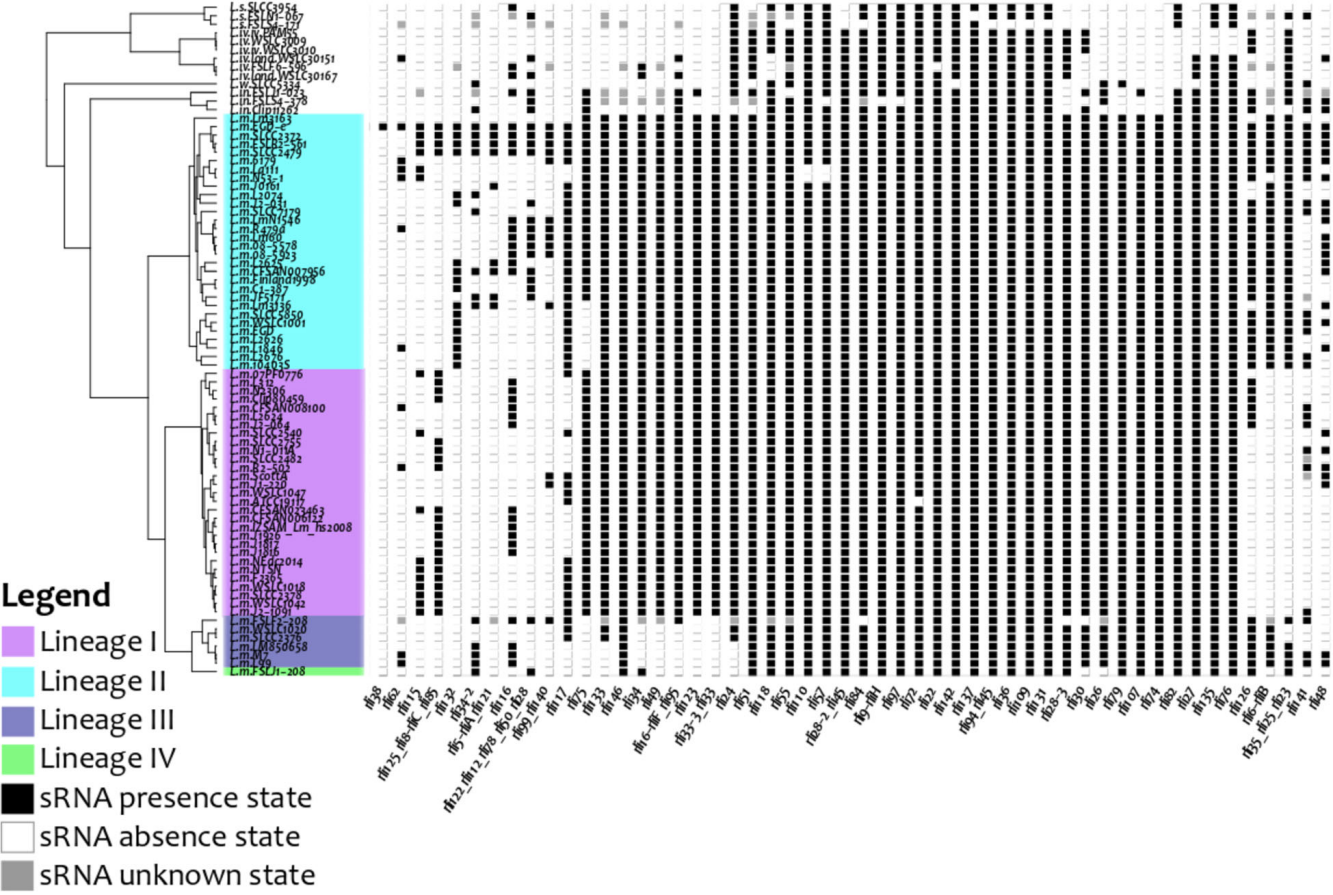
Phylogenetic distribution of *L. monocytogenes* EGD-e sRNAs in *Listeria genomes*. The figure represents the distribution of 52 variable L.monocytogenes EGD-e sRNA loci across 79 Listeria genomes. A black box indicates a presence (i.e. the sRNA sequence is present in the corresponding genome), while a white box indicates the absence (i.e. this sRNA sequence is not found in the corresponding genome). Listeria strains are ordered based on their placement in the Listeria reference tree shown on the left (see Fig. 2 for details)

Small-RNA presence-absence patterns along the *Listeria* phylogenetic reference tree are shown in Fig. 3. Most sRNAs are present in nearly all genomes, except mostly non-*L. monocytogenes* species. Small-RNA presence-absence patterns (Fig. 3) also suggest a link between sRNA content and previously defined *Listeria* lineages. For example most of sRNAs present in genomes of lineage I are also present in genomes of lineages II, but not systematically in lineage III and IV. Two sRNAs are found only in lineages I and II of *Listeria monocytogenes* (i.e., *see rli49* and *rli33-3_rli33*). Two other sRNAs are systematically absent of lineage I, while they are present in almost all the other *Listeria* genomes (e.g.*, rli6-rliB*, *rli23_rli25_rli35*). *Rli74* is specifically present in all four *L. monocytogenes* lineages and absent in other *Listeria* species. Additionally, several sRNAs exhibit sparse presence-absence patterns probably related to complex evolutionary histories.

### *Listeria* sRNA evolution and coevolution profiles

To investigate sRNA evolutionary histories, we inferred ancestral presence-absence patterns for all 52 variable sRNAs and obtained 44 different phyletic profiles (see Additional file 3: Table S3). Only eight sRNAs shared identical phyletic profiles as following: (1) *rli109, rli131, rli36* and *rli94_rli45*: one loss in branch 154 (*L. seeligeri/str.* FSL S4–171), (2) *rli9-rliH* and *rli97*: one loss in branch 137 (*L. innocua/str.* FSL S4–378) and (3) *rli135* and *rli76*: one loss in branch 147 (common ancestor of three *L. ivanovii* strains). This indicates that most profiles and evolutionary histories are specific to an sRNA, even if some evolutionary events are shared by several sRNAs. On the basis of the reconstructed sRNA gains and losses on the reference phylogenetic tree, we found only three sRNAs with monophyletic patterns (present in the most-recent common ancestor and all its descendants) (*rli146, rli38* and *rli74*) and two sRNAs with polyphyletic patterns (present in some genomes but not in their most-recent common ancestor) (*rli62* and *rli99_rli140*). All the other 47 (90%) sRNAs exhibit more or less complex paraphyletic patterns (present in the most-recent common ancestor and some of its descendants). Additionally, several sRNAs have undergone either a large number of gains (e.g., *rli116*: 10 gains, *rli115*: 5 gains) or a large number of losses (e.g., *rli122_rli112_rli78_rli94_rli50_rli28* locus: 13 losses; *rli141*: 10 losses; *rli26*, *rli48* and *rli117*: 8 losses) or both (e.g., *rli48*: 8 gains and 8 losses), suggesting that some sRNAs are subject to frequent reshuffle, even at short evolutionary scales.

The three monophyletic profiles indicate a scenario of gene appearance and descent. For instance, *rli146* and *rli74* exhibit the same monophyletic profile, i.e., one acquisition at branch 3 (ancestor of *L. monocytogenes* strains). It was inferred that *rli38* was acquired at branch 132 in *L. monocytogenes* EGD-e. Two different and complex polyphyletic patterns observed for *Rli62* and *rli99_rli140* suggest potential Horizontal Transfer events. All other sRNAs exhibit paraphyletic patterns, suggesting they underwent one or several loss events in the *Listeria* reference tree. Thirty-five out of 47 sRNAs (74%) with paraphyletic patterns are inferred to be present at the tree root, suggesting that the majority of sRNAs were present early, in *Listeria* evolution.

### The *Listeria* sRNA-coding gene coevolution network

We used Pagel’s model statistical framework [39] (see Methods) and both observed and ancestral presence/absence states to identify significant coevolution relationships between sRNAs and 5’UTRs/CDS regions along the *Listeria* reference tree. We obtained 23 putative sRNAs showing significant coevolutionary relationships with 23 5’UTRs and 39 CDS of 52 coding genes (see complete list in Additional file 4: Table S4).

**Fig. 4:**
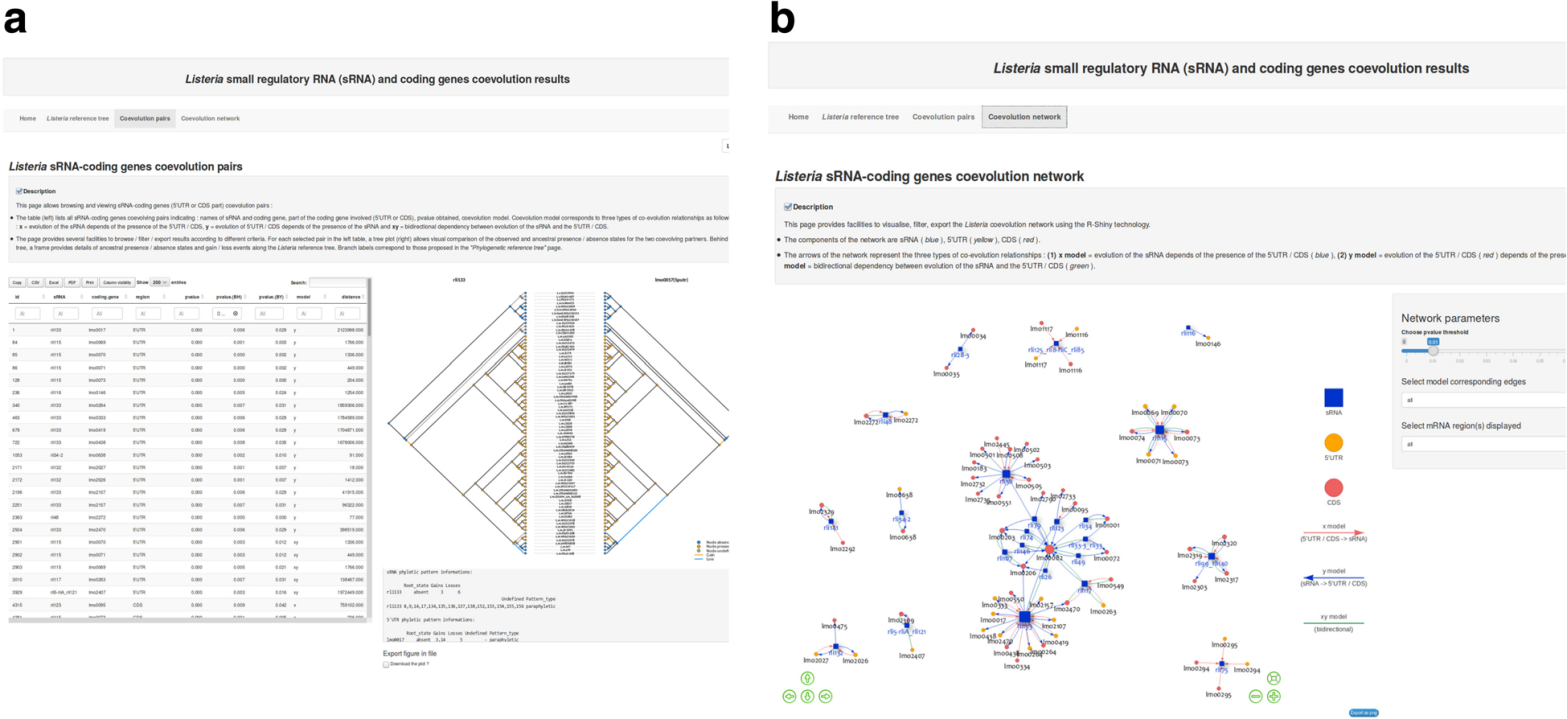
*Listeria* sRNA, 5’UTR and CDS evolution and coevolution results. **a** The Listeria sRNA, 5’UTR and CDS coevolution results available on the Shiny web site. The left frame allows the browsing of the results and the selection of coevolving pairs. The right frame allows visualization of phyletic patterns (i.e. observed and ancestral presence/absence patterns) on the Listeria reference tree for each selected pair. **b** The Listeria coevolution network available on the web site. The network represents predicted coevolution between *L. monocytogenes* EGD-e sRNAs and either 5’UTRs or CDS regions (compared to the null hypothesis of independent evolution between these elements). sRNAs are indicated in blue while 5’UTRs and CDS are indicated respectively in yellow and red. The arrows of the network represent three types of coevolution relationships: (i) evolution of the sRNA depends on the presence of the 5’UTR/CDS (blue) (ii) evolution of 5’UTR/CDS depends on the presence of the sRNA (red) and (iii) bidirectional dependency between evolution of the sRNA and the 5’UTR/CDS (green)

All results of sRNAs, 5’UTRs and CDS phyletic patterns, coevolution analyses and the resulting coevolution network were made available on a dedicated web server that provides several facilities to browse the results: http://genoweb.toulouse.inra.fr/Listeria_sRNA. The web application was developed with the *Shiny* technology [41] and allows interactive visualization of individual phyletic patterns (i.e., observed and inferred ancestral presence/absence patterns) for all sRNAs and their coevolution partners along the *Listeria* reference tree (see an example in Fig. 4).

### The *Listeria* sRNA-coding gene coevolution hub

The inferred sRNA-coding genes coevolving network (see Fig. 4b) reveals interesting features. We observed a hub of 12 sRNAs (*rli107*, *rli117*, *rli123*, *rli133*, *rli146*, *rli26*, *rli30*, *rli33-3_rli33*, *rli34*, *rli49*, *rli74* and *rli79*) that are connected through common coevolution partners. This cluster includes mainly distant (i.e. distance >40 kb) 5’UTRs and CDS coevolving partners, with the exception of partners of only two sRNAs: *rli30* paired to CDS partners *lmo0501* to *lmo0508,* and *rli74* with its CDS partner *mpl* (*lmo0203)*. This cluster includes many genes encoding cell wall proteins, proteins involved in secondary metabolism and virulence factors (see next section and *rli133* case study for details). The other 11 sRNAs are all included in 11 individual clusters that contain either 5’UTR coevolving regions (*rli132*, *rli116*) exclusively or CDS coevolving regions (*rli28–3*, *rli99_rli140*, *rli141*) exclusively or a mix of both 5’UTRs and CDS regions (*rli75*, *rli5-rliA_rli121*, *rli34–2*, *rli115*, *rli125_rli8-rliC_rli85*, *rli48*). Interestingly, nine out of 11 of these individual clusters include evolving partners that are close on the genome (< 8 kb). Two individual coevolving groups [*rli28–3*/*lmo0035*] and [*rli5-rliA_rli121*/*lmo2309-lmo2407*] were found with distant coevolution partners (distance >800 kb for both clusters). To summarize, our results reveal an sRNA hub including mainly distantly located coevolving 5’UTRs and CDS regions, some of them exhibiting functions related to *Listeria* pathogenicity. Most of the remaining coevolution clusters include pairs of sRNA and 5’UTRs/CDS that exhibit very close genomic positions, i.e. a distance between the start of their sRNA and the start of their 5’UTR/CDS under 8 kb.

We investigated the functional classes of genes coevolving with *Listeria* regulatory sRNAs by using annotations from the Clusters of Orthologous Groups (COGs) database [42] retrieved from the Listeriomics website [36]. The distribution of coding genes in COG categories reveals a significant functional enrichment of coding genes associated with cell wall or membrane biogenesis (see Table 1, Fisher exact test [43], *p*-value = 0.0131). Interestingly, among coding genes coevolving with *Listeria* sRNAs, we found seven internalin genes (out of an expected 26 in Listeria [36]), two coding genes of the *Listeria* Pathogenicity Island LIPI-1 *mpl* (lmo0203) and *orfX* (lmo0206) [44, 45], one component of the flagellar biosynthesis pathway, eight genes involved in secondary metabolism and bacteriophage genes (see Additional file 4: Table S4 for details).

**Table 1:** Functional enrichment of sRNAs and coding genes coevolution groups

Functional category	*P* value
Amino acid transport and metabolism	0.9055
Carbohydrate transport and metabolism	0.2202
Cell wall/membrane biogenesis	0.0131*
Energy production and conversion	0.8563
Replication, recombination and repair	0.8610
Secondary metabolites biosynthesis, transport and catabolism	0.5206
Signal transduction mechanisms	0.3669
Transcription	0.2648

This table contains p-values obtained with Fisher tests to measure a potential enrichment of a COG functional category in coding genes found to co-evolve with sRNAs (Additional file 4: Table S4). The * indicates a significant (under 0.05) p-value for genes of the category Cell Wall/membrane biogenesis

### rli133, an sRNA coevolving with genes known to be involved in pathogenicity

**Fig. 5:**
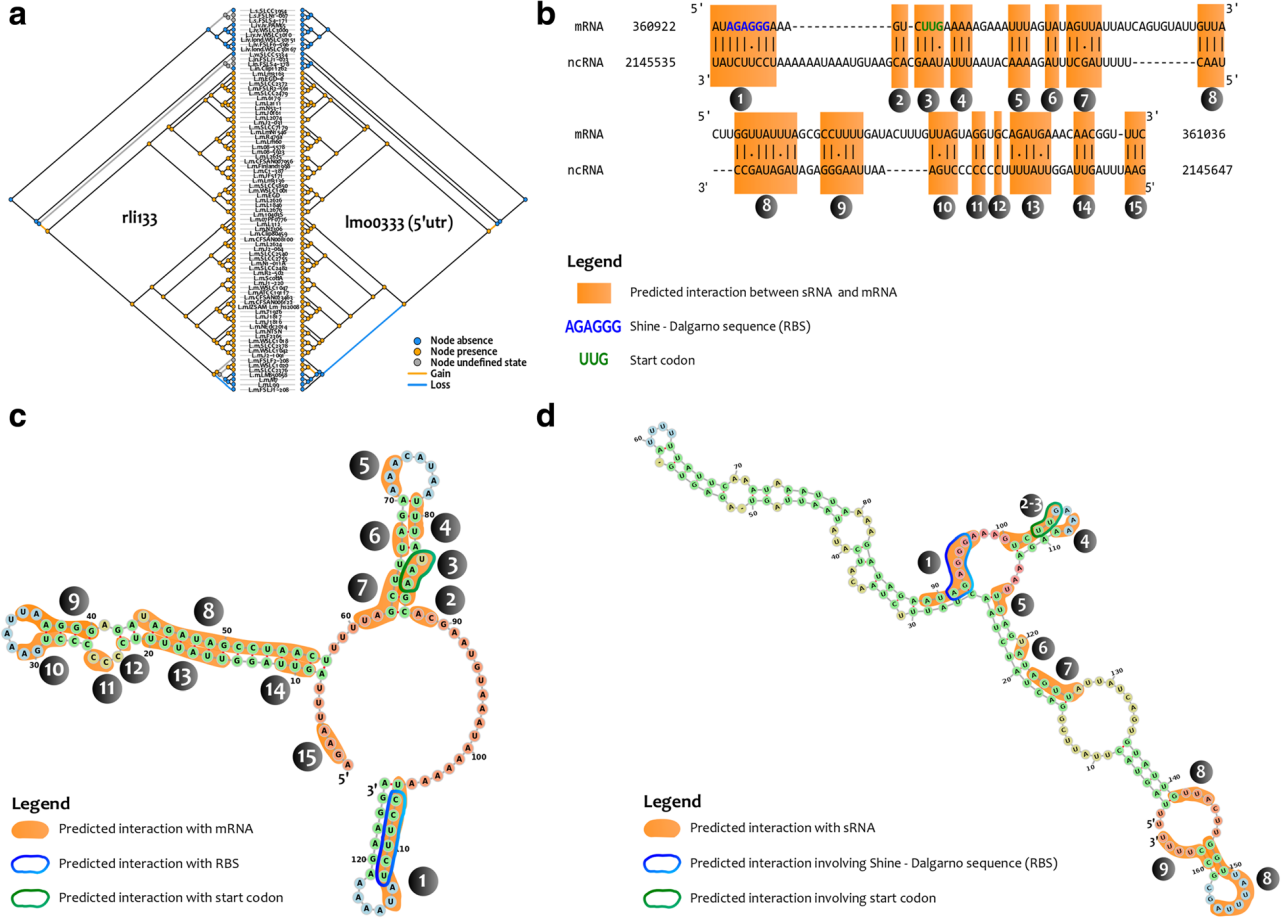
The *rli133* coevolution ties. rli133 shows significant coevolution with six CDS and nine 5′-UTR regions. Figures 5a to 5d show an example of a coevolution pattern and a putative mechanism of interaction between rli133 and the lmo0333 5’UTR region corresponding to the promoter of an Internalin IntI protein. **a** Coevolution patterns observed between rli133 (left) and the 5’UTR of lmo0333 (right). Yellow circles correspond to observed (or ancestral) sRNA/CDS presence. Blue circles correspond to observed (or ancestral) sRNA/CDS absence. A yellow branch indicates a sRNA/CDS gain event while a blue branch indicates a loss event along the branch. **b** Predicted interaction regions between rli133 and lmo0333. The figure presents the interaction regions between the 5’UTR (and the beginning of the coding region) and the sRNA. Highlighted and numbered regions correspond to predicted interaction zones according to the RNAplex software. **c** Predicted structure of the sRNA rli133. Structure was generated using LocaRNA software and a multiple alignment of all conserved rli133 sRNAs in the genomic dataset. Highlighted numbered regions correspond to Lmo0333–5’UTR predicted interaction zones of Fig. 5b. Representation of structure was performed with FoRNA. **d** Predicted structure of the 5’UTR region of Lmo0333. Structure was generated using LocaRNA and a multiple alignment of all conserved Lmo0333 5’UTRs in the genomic dataset. Highlighted numbered regions correspond to rli133 predicted interaction zones of Fig. 5b. Representation of structure was performed with FoRNA

The detailed analysis of *rli133* phyletic pattern (Fig. 5a) reveals an early acquisition event in *L. monocytogenes* common ancestor. *Rli133* cannot be found in other *Listeria* species, indicating that this sRNA is specific to *L. monocytogenes* species. Nevertheless, *rli133* is lost in four strains of lineage III (*L. monocytogenes FSLF2–208*, *L. monocytogenes LM850658, L. monocytogenes M7, L. monocytogenes L99*) and in the *L. monocytogenes FSLJ1–208* strain of lineage IV. In these five strains, the corresponding intergenic region is missing due to the insertion of two genes. These genes appear to be specific of these five strains and do not have any homolog in public databases (see Additional file 5: Figure S1). In genomes where *rli133* is present, the corresponding sequence is well-conserved and includes few mutation events, i.e., six transitions, two transversions and two indels events corresponding to 12 variables sites out of 126 (9.6%) in the *rli133* alignment (see Additional file 6: Figure S2). Considering these mutated sites, *rli133* homologous sequences can be easily separated in two clusters that correspond to *Listeria* lineage I and II. *Rli133* presents coevolutionary relationships with 12 coding genes, eight 5′ UTRs and seven CDS regions. Coevolving gene partners include three internalin genes: *inlI* (*lmo0333*), *inlE* (*lmo0264*) and *inlP* (*lmo2470*). Internalins are important virulence factors [46, 47]. *InlE* may contribute to host tissue colonization [46, 48] and *InlP* has recently been shown to promote placental infection [47]. The role of *inlI* in pathogenicity remains to be determined [49]. Interestingly, we found three other sRNAs potentially coevolving with internalin genes: *rli117* (*lmo0549*, *lmo0263* and *lmo2470*), *rli30* (l*mo2445*) and *rli132* (*lmo2017*). Other genes were found to coevolve with *rli133*, e.g.*, lntA, lmo0206* and *sepA.* The virulence factor *lntA* (*lmo0438*) targets the chromatin repressor *BAHD1* to activate interferon-stimulated genes in the host cell nucleus [50]. Expression of *LntA* seems to be tightly controlled to subvert immune responses and prevent antibacterial responses [50]. The *orfX* (*lmo0206)* gene is located within the *L. monocytogenes* pathogenic island 1 (LIPI-1), which includes genes required for *Listeria* intracellular lifestyle such as *hly, plcA, plcB and actA* [33] and contributes to bacterial survival in macrophages. *SepA* (*lmo2157*) encodes a protein involved in septum formation and play a role in stress response [51]. To summarize, six out of the twelve coevolution partners of *rli133* exhibit a documented function linked to either pathogenicity, interaction with host cells or stress response [36, 46, 47, 51]. Moreover, *rli133* sRNA was found to be expressed in several transcriptomes during infection, especially in blood and intestine [5, 22, 36]. Coevolution between sRNAs and coding genes may be resulting from the existence of direct or indirect functional links. Direct functional links can be explained by physical interaction through base-pairing at specific regions of a sRNA with their target mRNA. To identify possible physical interaction between *rli133* and its coevolution partners, we used several methods to predict structure of both the sRNAs and the 5’UTRs/CDS interacting regions and look for putative interacting zones (see Methods).

We found regions possibly interacting with *rli133* for all the 12 genes coevolving with *rli133*. Nine of these genes were identified to present interacting regions compatible with a negative regulation mechanism: *inlI* (*lmo0333*), *inlE* (*lmo0264*), *inlP* (*lmo2470*), *lntA* (*lmo0438*), *orfX* (*lmo0206*), *lmo0082*, *lmo0334*, *lmo0550 and lmo2107*. For the three remaining coevolving genes (*lmo0419, lmo0017* and *lmo2157*) we did not identify a consistent interacting region. As illustrated in Fig. 5b, fifteen interacting regions were predicted between *lmo0333*–5’UTR and *rli133*. Two interacting regions were overlapping in the proximity of the Shine-Dalgarno sequence and the initiation codon (see regions 1 and 3 in Fig. 5b, c and d), which are crucial sites for ribosome recruitment during the initiation of the translation. On the mRNAs, these sequences are mainly found to be accessible in loops or pseudo-loops (Fig. 5d), suggesting that they are constitutively available for translation. Interaction with complementary regions on sRNAs potentially makes them unavailable for ribosome binding during translation initiation, suggesting a potential inhibitory action of *rli133* at posttranscriptional level on these genes. The existence of direct interaction between *rli133* and coevolving genes partners could explain their coevolution.

To conclude, interacting regions corresponding to putative translation inhibition regions targeted by rli133 were identified for nine coevolving genes including *inlE* [46, 48], *inlI* [49], *inlP* [47], *lntA* [50] and *orfX* which were already found to be involved in host-interaction in previous studies.

## Discussion

We built a robust workflow that provided new insights on *Listeria* sRNA evolution and coevolution patterns. First, the screening of sRNA presence-absence patterns suggests that 60 out of 112 *L. monocytogenes* EGD-e sRNAs (53%) shape the *Listeria* sRNA core set, in the sense that they were found to be present and conserved in all *Listeria* genomes. These 60 sRNAs were hence inferred present in the common ancestor of *Listeria*, suggesting that they were present early during the evolutionary history of the genus. This is a lower proportion compared to the 60 out of 83 *E. coli* K12 sRNAs (72%) that were found present and conserved in a dataset of 27 complete genomes of *E. coli-Shigell*a [29]. However, this is consistent with the higher number of genomes and the wider evolutionary scale (genus) used in our analysis compared to the species level of the *E.coli-Shigella* study.

The 52 remaining *L. monocytogenes* EGD-e sRNA loci constitute the variable sRNA set that is part of the *Listeria* accessory genome. This number is higher than the 43 accessory *Listeria* sRNAs previously identified by Kuenne et al. [38] in a smaller dataset of 11 genomes restricted to *L. monocytogenes* species. We found a higher proportion of them exhibiting complex paraphyletic distribution compared to the *E. coli/Shigella* study: 47 variable sRNAs out of 52 were shown to have paraphyletic pattern (90%) compared to 25 out of 32 (78%) in the 27 genomes of *E. coli-Shigella* [29]. Only three and two sRNAs have monophyletic and polyphyletic patterns, respectively. This indicates complex and various evolutionary histories underlying diverse origins and a potentially wide panel of sRNA acquisition and loss mechanisms in *Listeria*.

Detection of coevolution and analysis of the *Listeria* sRNA-coding genes coevolving network highlighted many interesting features.

We revealed an evolutionary link between sRNAs and coding genes related to pathogenicity and interaction with the host cell that suggests a key role for these sRNAs to shape *Listeria* virulence and adaptation. More precisely, we identified a hub of 12 sRNAs (*rli26, rli30, rli33-3_rli33 locus, rli34, rli49, rli74, rli79, rli107*, *rli117*, *rli123*, *rli133* and *rli146*) coevolving with many genes encoding cell wall proteins, especially internalins, that are known to be involved in host cell interaction [52], proteins involved in secondary metabolism, stress response and virulence factors. We detected a significant coevolution pattern of four sRNAs *(rli117*, *rli30*, *rli132* and *rli133*) and seven internalin genes (*lmo0549, lmo0263, lmo2470, lmo2445, lmo2027, lmo0333 and lmo0264*), indicating a probable key functional role of these sRNAs on these genes, possibly regulatory. To our knowledge, the relationship between small regulatory RNAs and internalin evolution was never observed before and opens several new perspectives concerning the possible impact of sRNAs in *Listeria* evolution and virulence. These results are consistent with previous observations that several internalin genes present long 5’UTRs that may also be post-transcriptionally regulated and that *Listeria* controls many of its virulence genes by a mechanism that involves 5’UTRs [23].

Interestingly, previous studies performed in *E. coli* or *S. enterica* have shown that sRNAs are often found to control the expression of cell wall proteins, particularly in outer membrane [53] or lipopolysaccharide layer synthesis [54]. This is consistent with our result revealing that the ‘cell wall or membrane biogenesis’ functional category is significantly overrepresented in *Listeria* sRNAs coevolving genes. Namely, we found seven internalin genes and two coding genes of the *Listeria* Pathogenicity Island LIPI-1 (*mpl*, *orfX*) in the *Listeria* sRNA coevolution partners. These results suggest a possible key regulatory role of some *Listeria* sRNAs on genes involved in host-bacteria interaction and pathogenicity.

We focused on *rli133*, a *L. monocytogenes*-specific sRNA, and identified 6 out of 12 *rli133* coevolution partners exhibiting a function linked to either pathogenicity, interaction with the host cells or stress response. Interacting regions compatible with mechanisms of mRNA translation inhibition were predicted for *rli133* and nine coevolving genes, including *inlE* [46, 48], *inlI* [49], *inlP* [47], *lntA* [50] and *orfX*. These results suggest a possible direct negative regulatory role of *rli133*, which potentially impairs the translation process of some of its coevolving partners. The presence of compatible interacting regions is not a feature specific to genes coevolving with *rli133*, but taking together the observations of coevolution pattern and the presence of a consistent interacting zone argue in favor of a functional link. Moreover, we looked for the presence of the nine 5’UTR-interacting zones of the genes co-evolving with rli133 in 5’UTRs and CDS that do not coevolve with rli133 and found only two similar regions for the inlP (lmo2470) interacting zone: one located in another internalin 5’UTR region (inlP/lmo2027) and one located in the 5’UTR region of the lmo0974 gene that is involved in LPS synthesis and conserved in all the genomes of the dataset. This argues for quite a good specificity of the *rli133* predicted interacting zones. For coevolving partners in which no clear mechanism were highlighted, such as *sepA* (*lmo2157*) [51], a well-known stress response gene involved in septum formation, coevolution patterns may correspond to presence of direct interaction at post-translational level or indirect functional links involving intermediate genes. These results suggest that *rli133* could act as a negative regulator of genes involved in *Listeria* pathogenicity.

Interestingly, rli133 sRNA is missing in the *L. monocytogenes* M7 and *L. monocytogenes* L99 genomes of *L. monocytogenes* lineage III that also have a reduced internalin-coding genes content (respectively 17 and 18 internalins) [55–57]. This suggests a possible link between the presence of *rli133*, the internalin gene content and the regulation of pathogenicity. The situation may be more complicated in other genomes such as the pathogenic strain *L. monocytogenes* J1–208 (lineage IV) identified in goat and whose chromosome contains only 16 internalin-coding genes and no *rli133* sRNA. This strain includes a plasmid (pLMIV) which contains additional internalins that may be involved in another mechanism of regulation of pathogenicity [57]. This indicates that the presence of *rli133* is not an absolute hallmark for pathogenesis and that other, yet unannotated sRNAs may interact with internalin genes in pathogenic strains of lineage IV. Additional genomes and sRNA experimental datasets are needed in this clade to fully understand the role of sRNAs and internalin coding genes in *Listeria* pathogenicity.

The *Listeria* coevolution network also pointed out 11 sRNAs exhibiting correlated evolution, mostly with close 5’UTRs and CDS regions. Screening for distances between coevolving sRNAs and genes indeed revealed two trends concerning gene location: on one hand, genes close to the corresponding sRNA (putative *cis*-regulated genes closer than 8 kb), and on the other hand, genes found at distant locations (putative *trans*-regulated genes with distances higher than 40 kb) (see Additional file 7: Figure S3). One possibility is that some of the closer coevolving sRNAs may correspond to uncharacterized or unannotated 5’UTR regions.

## Conclusion

The analysis of the *Listeria* coevolving network sheds light on several sRNAs which might play a role in virulence regulation. Since our approach makes it possible to obtain a list of sRNAs present only in the virulent strains, this study paves the way for new biochemical and biological analyses aimed at identifying and deciphering new factors involved in virulence.

The workflow proposed in this work is resourceful and, to our knowledge, does not have any equivalent in previous work. Our strategy proposes several methodological enhancements and additional analyses compared to the pioneer work of Skippington and Ragan [29]. For instance, our strategy was designed to deal with uncovered regions of draft genomes and paralogy problems (both for sRNAs and coding genes). Moreover, three key steps of our workflow, i.e. the reference tree construction, the inference of ancestral presence-absence states and the detection of coevolution between sRNAs and coding genes, rely on the statistical framework of continuous-time Markov models and maximum likelihood, improving on parsimony approaches that do not provide consistent branch length estimation and may lead to lower precision.

Another key advantage of our approach is its extensively generic character since it can be transposed to any type of organism, any type of functional data and, more generally, to any kind of qualitative trait. For example, the strategy developed may be used to look for a possible coevolution between regulatory or structural RNAs and any type of element or feature such as pathogeny islands, pseudogenes, CRISPRs, phages, insertions sequences, etc. The entire workflow is built on an open source frame that is flexible, optimized and implements parallel and distributed computation, while however remaining computationally demanding.

Several features may be proposed in the future to enhance the proposed strategy. First, as we currently only consider presence, absence and unknown states, it constitutes an oversimplification of the way functional elements are defined, also undermining paralogy for sRNA or coding genes. Consequently, an enhancement of our strategy would be to deal with the occurrence of sRNAs and coding genes for both evolution and coevolution analysis. Second, another useful extension of the current strategy would be to include the analysis of mutation patterns and coevolving sites also for the core sRNAs and coding genes present in all the genomes of the dataset as well. This could be performed by including an additional step in the workflow that relies on a previously published method like the CoMap software [58].

## Methods

### *Listeria* genome dataset

Seventy-nine complete public genomes of *Listeria* were obtained from GenBank (release 211). A full description of the 79 *Listeria* genomes is available in Additional file 1: Table S1). The dataset includes 70 complete and 9 draft genomes representing five different *Listeria* species (*L. monocytogenes, L. ivanovii, L. inoccua, L. welshimeri* and *L. seeligeri*). *L. monocytogenes* genomes were the most represented (73 genomes corresponding to 92% of our dataset).

### *Listeria monocytogenes EGD-e* sRNA

A set of 304 experimentally validated sRNAs from *L. monocytogenes* EGD-e was extracted from the Listeriomics database [36]. We focused on the 154 sRNAs annotated as putative trans sRNAs which are known as important regulators of gene expression in bacteria acting on independently expressed targets. A group of 19 sRNAs were excluded because they recently have been found to include small ORFs [6]. Overlapping sRNAs and sRNAs harboring paralogs in the *L. monocytogenes* EGD-e genome were processed using the following procedure. sRNAs were aligned on the *L. monocytogenes* EGD-e genome sequence using BLASTN+ [59]. Overlapping hits were merged considering a minimal overlap length of 15 pb, independently of their orientation. Finally, 112 sRNA loci were used as input sequences in the following analyses, including 15 loci built from merged hits and 97 original sequences.

### sRNA and coding gene coevolution strategy

The strategy we developed is implemented in a Snakemake workflow [60] that consists in four main steps (see Fig. 1).

Step 1: Phylogenetic reference tree.

PanOCT, version 3.23 [61], was used to build groups of orthologs from annotated genomes. PanOCT is able to deal with recently diverging paralogs by using neighborhood gene information (synteny). All the parameters were set to default values except for the length ratio to discard shorter protein fragments when a protein is split due to a frameshift or other mechanisms was set to 1.33 as recommended by the authors. Amino-acid sequences of ortholog families were then aligned using ProbCons, version 1.12 [62], and resulting alignments were post-processed using GBLOCKS, version 0.91b [63], using the following parameters: the minimum number of sequences for a conserved position was set to (n/2) + 1, the minimum number of sequences for a flank position to (n/2) + 1 (where *n* is the total number of sequences in the aligned dataset), the maximum number of contiguous non-conserved positions was set to 20, the minimum length for a block to 5, and gap positions were allowed [8].

The reference tree was built using the syntenic core gene families corresponding to the PanOCT clusters with a single unique ortholog in each genome of our dataset. The corresponding nucleic acid alignments were obtained from all these core families filtered amino-acid alignments and concatenated into a single superalignment to compute a maximum likelihood tree using FastTree2, version 2.1.9 [64]. The following parameters were used for FastTree2: the Generalized Time-Reversible model (GTR) was chosen, the likelihood was reported under the Gamma model using 20 categories of sites, the exhaustive search mode (“-slow” option) was selected to obtain a more accurate reconstruction, NNI and SPR heuristics were used to browse the tree space. Support analyses were performed using Shimodaira Hasegawa test (SH) and 1000 resampling steps of site likelihood.

Step 2: sRNA and coding gene presence-absence matrix.

Presence-absence patterns were inferred from BLAST analyses with different parameters for sRNAs and coding genes. *L. monocytogenes* EGD-e sRNAs were aligned on the genome dataset using BLASTN+, version 2.2.29 [59]. Resulting hits were filtered using two criteria: an e-value <10^−2^ and a coverage related to the query sequence ≥70%. Only sRNAs meeting these two criteria were considered as present in the targeted genomes.

For coding genes, we analyzed separately 5’UTRs and CDS regions. *L. monocytogenes* EGD-e CDS were retrieved from GenBank annotations and aligned against all Listeria genomes using BLASTP+, version 2.2.29 [59]. Resulting hits were filtered using the following criteria: an e-value <10^−2^, a coverage relative to the query sequence ≥70%, an identity rate ≥ 60% and a bitscore ≥50. Only CDS meeting these three criteria were considered as present in the targeted genomes.

*L. monocytogenes* EGD-e 5‘UTRs were retrieved using the following procedure. When available, we used experimental data indicating 5’UTR positions [36] to extract the corresponding DNA sequence. When not available, 5’UTR positions were defined arbitrarily as the 100 nearest 5’ nucleotides upstream from each *L. monocytogenes* EGD-e CDS start codon of intergenic region. Only 5’UTRs with a minimum size of 15 bp were kept. 5’UTR sequences were then used as queries for BLASTN+ [59] alignments against all genomes. Resulting BLASTN+ hits were filtered using the following criteria: an e-value <10^−2^, and a minimal identity percentage and coverage adjusted to the 5’UTR sequence lengths as follows. For 5’UTRs with lengths from 15 to 20 bp, the minimum identity percentage was set to 90% and the minimum coverage percentage to 100%. For 20–50 bp long 5’UTRs, both identity percentage and coverage were set to a minimum of 80%. For 50–100 bp and >100 bp long 5’UTRs, the minimal identity percentage was set to 80% and the minimal coverage was set to 50% and 25%, respectively. 5’UTRs meeting these criteria in subject genomes were considered as present.

A binary vector (0/1) corresponding to the absence/presence profile in the whole genome dataset was finally generated using BLASTN+ results and filters defined above. Due to their lack of informative value, sRNAs, 5’UTRs and CDS found in all genomes were not taken into account in subsequent analyses.

To avoid absence mispredictions corresponding to unsequenced regions of draft genomes, absence events of non-coding elements (sRNAs and 5’UTRs) were systematically checked as follows: for queries without hit in a given genome, 5′ and 3′ adjacent genes of the query element were screened for putative homologs in the same genome. In case where homologs were found, the non-coding sequence between the two homolog genes was extracted and screened for stretches of Ns, i.e. assembly gaps. If such stretches were found, the state of the query element was considered as undetermined due to missing DNA region in the considered genome (‘?’ state assigned). If the region was present but not similar to the query sequence, the query element was consider to be absent (‘0’ state assigned).

Step 3: Ancestral presence-absence pattern reconstruction.

Presence/absence ancestral states were reconstructed using the recent “Hidden rates model” method proposed by Beaulieu et al. [65]. This method uses Hidden Markov Models (HMM) to reconstruct ancestral character states from observed states and a reference phylogenetic tree. It makes it possible to use different transition rate classes. We used the ‘*rayDISC*’ function of the ‘*corHMM*’ R package version 1.20 [17] and selected the *‘ARD’* transition model, i.e. independent transition rates. Internal node states were inferred using maximum likelihood estimation and joint probabilities. The root state probabilities were inferred using the method of Fitzjohn and Maddison [66]. Initial transition rates were estimated using the results of PanOCT orthologs obtained in Step 1 and computed using the ‘*DiscML*’ function of the ‘*DiscML*’ R package, version 1.0.1 [67], and the ‘*ARD*’ transition model (assuming independent transition events, in this case, gain and loss events, for each element). This step results in a matrix containing the binary presence/absence (0/1) pattern for each internal node of the reference tree and for the three analysed features (sRNAs, CDS, 5’UTRs). Finally, gain and loss events were determined as follows: if the feature was absent (present) in a given node but present (absent) in its ancestor, it was considered as lost (gained) along the corresponding branch linking both nodes.

Step 4: Detection of coevolution events.

Our strategy allows the detection of coevolution between a sRNA and a 5’UTR or CDS using a reference phylogenetic tree and both observed and ancestral presence-absence patterns. We used the ‘*corDISC*’ function of the ‘*corHMM*’ R package [65] to identify putative coevolutionary relationships. This function fits Pagel’s models of independency and dependency [39] to identify dependent evolution between two binary characters (in this case, the presence or absence of sRNAs, CDS/5’UTRs) and related to a phylogenetic tree. The first model supports an independent relationship between both binary traits: sRNA and 5’UTR/CDS (the null hypothesis). The second kind of model (the alternative hypotheses) supports a dependent relationship between both traits (coevolution). The use of ancestral states along the phylogenetic reference tree makes it possible to evaluate the probable temporal ordering of changes between two x and y presence/absence patterns and to test hypotheses about cause and effect. For this, we used three kinds of dependency models: the *x* model, meaning that the evolution of the sRNA depends on the presence/absence state of the 5’UTR/CDS, the *y* model, meaning that the evolution of 5’UTR/CDS depends on the presence/absence state of the sRNA and the *xy* model, assuming bidirectional dependency between evolution of the sRNA and the 5’UTR/CDS element.

The ‘*corDISC*’ function merges the two x, y traits in a single vector, fits them on a precomputed phylogenetic tree using a specified model and then returns the likelihood of the model. The likelihood of each model was computed and a Likelihood Ratio Test (LRT) was performed. The corresponding *p*-value was computed. All of the analyses were performed between each variable sRNA, each variable CDS and 5′ UTR. *P*-values were corrected for multiple testing using the Benjamini-Hochberg (BH) procedure [68]. Finally, we only retained coevolving pairs of sRNA loci and coding gene elements (5’UTR and CDS) with a minimum BH corrected *p*-value threshold of 0.01.

The coevolution network between sRNAs and coding genes was reconstructed using inferred significant dependency relationships between phyletic patterns of sRNAs and coding gene elements (5’UTR and CDS) of *L. monocytogenes* EGD-e. Graph representations were built using the ‘*igraph*’, version 1.0.1 [69], ‘*visNetwork’*, version 1.0.3 [70], and ‘*Shiny’*, version 1.0.1 [41] R packages.

### Gene targets functional enrichment

Functional enrichment test was performed using *L. monocytogenes* EGD-e gene COG ontologies [36] and computed using a Fisher’s exact test [43] (‘*fisher.test’* function from the ‘*stats’* R package, version 3.5.0 [71]), with a *p*-value threshold of 0.05.

### Interacting regions prediction

Possible physical interactions between sRNAs and coding genes identified as coevolving partners using our method were predicted using several pieces of software: Ssearch (implementation from Wisconsin Package), version 6.1, IntaRNA, version 2.0.2, RIsearch, version 1.1, RNAcofold, version 2.3.4 and RNAplex, version 2.3.4 [72–76], which are all included in the sRNAtabac resource [77]. We used an extended region including 100 bp before and after the start codon to identify putative interactions between a sRNA region and the extended 5’UTR region of mRNAs (original regions were used for CDS). Only interactions containing a minimum of six successive interacting matches were selected and considered as valid.

Homologous sequences of rli133 previously identified (see Step 2 for details) in *L. monocytogenes* genomes were extracted. Homologous sequences of lmo0333 (inlI) 5’UTR (see Step 2 for details) were extended up to 60 nucleotides after the start codon. *Rli133* and *lmo0333*-extended 5’UTR sequences were processed using LocARNA software, version 1.8.9 [73]. LocARNA is a tool that allows simultaneous folding and alignment of input RNA sequences. LocARNA default alignment accuracy was increased using match probabilities and probabilistic consistency transformation. Additional parameters were used since it is recommended by the authors in the software documentation for aligning up to about 15 sequences of lengths up to a few hundred nucleotides. The weight of base pair match contribution was set to 400. An iterative refinement of the progressive alignment was performed using two iterations. The 2D structure representation of *rli133* and *lmo0333*- extended 5’UTR were computed with FoRNA, version 0.1 [78], using consensus structures of *rli133* and the *lmo0333*-extended 5’UTR associated with corresponding sequences of the reference strain *L. monocytogenes* EGD-e.

## Additional files

Additional file 1: Table S1. List of the 79 genomes used in this study. The table includes the list of 79 genomes obtained from Listeriomics and retrieved from the NCBI database. Several fields have been abbreviated for easier reading: ‘Se.’: strain serotype, ‘Li.’: Listeria lineage and ‘Co.’: country where the strain was first isolated. (DOCX 98 kb)

Additional file 2: Table S2. List of the 112 sRNA loci used in this study.The table includes 97 sRNAs obtained from the Listeriomics database and 15 merged regulatory sRNA loci tagged with an * in the table and obtained from the procedure described in Methods. (XLSX 21 kb)

Additional file 3: Table S3. Ancestral presence/absence patterns of L.m. EGD-e regulatory sRNAs. For all 52 L.m. EGD-e variable sRNAs, the table includes the following information according to the Listeria reference tree (see Fig. 2): root presence - absence information (Root state column), tree branches labels where gain (Gains column) and loss events (Losses column) were inferred (labels correspond to branch identifiers indicated in the cladogram of Fig. 2). The undefined column corresponds to tree branch labels with undefined state due to missing data in the corresponding genomes (draft genomes). The pattern_type column corresponds to the three different types of phyletic profiles inferred: monophyletic, polyphyletic or paraphyletic profiles. (DOCX 128 kb)

Additional file 4: Table S4. Listeria sRNAs and coding genes coevolution groups. For each sRNA, the table includes the following informations on the corresponding co-evolving elements: the gene locus tag name (‘Element’ column), the type of element (CDS or 5’UTR, ‘Type’ column), the type of dependency model that highlighted the interaction: x = evolution of the sRNA depends on the state of the 5’UTR/CDS, y = evolution of 5’UTR/CDS depends on the state of the sRNA and xy = bidirectional dependency between evolution of the sRNA and the 5’UTR/CDS element (Model column), the distance between the sRNA and the element in nucleotides (Distance column) and the description of the gene/operon function according to Listeriomics database (‘Description’ column); ‘id’ = identical content. Coevolution groups that are included in the main network hub are highlighted in gray. (DOCX 142 kb)

Additional file 5: Figure S1. Rli133 genomic context conservation in Listeria. 5′ and 3′ homolog genes are represented using red arrows. GFXXXX names correspond to PanOCT ortholog clusters identifiers. Blue arrows correspond to two genes inserted in several strains of Listeria lineages III and IV. (PDF 212 kb)

Additional file 6: Figure S2. Multiple alignment of rli133. This figure represents the multiple alignment of rli133 sequences in strains where it is present. Red denotes a fully conserved position. The phylogenetic tree at the left corresponds to a Maximum Likelihood tree computed from the corresponding multiple alignment. (PDF 1413 kb)

Additional file 7: Figure S3. Genomic distance between coevolving sRNAs and CDS. Plain curves show the distance density between sRNAs and 5’UTRs (red) or CDS (blue) engaged in coevolution relationships, considering genome circularity. They are compared to distances between sRNAs and all 5’UTRs or CDS (all) respectively represented by red and blue dotted curves. (PDF 117 kb)

## Abbreviations

5′ UTR: 5′ Untranslated Region of coding genes
CDS: Coding DNA Sequence
COG: Clusters of Orthologous Group
mRNA: messenger RNA
SH: Shimodaira Hasegawa
sRNA: Small regulatory RNA
