# Supplementary material for: Unraveling the evolution and coevolution of small regulatory RNAs and coding genes in Listeria
Source: BMC Genomics. 2017 Nov 16;18:882. doi: 10.1186/s12864-017-4242-0 (PMC5689173; doi:10.1186/s12864-017-4242-0)
Supplement: Supplementary file 1 — List of the 79 genomes used in this study. The table includes the list of 79 genomes obtained from Listeriomics and retrieved from the NCBI database. Several fields have been abbreviated for easier reading: ‘Se.’: strain serotype, ‘Li.’: Listeria lineage and ‘Co.’: country where the strain was first isolated. (DOCX 98 kb) [file 12864_2017_4242_MOESM1_ESM.docx]

| **Accession** | **Spec/Strain** | **Se.** | **Li.** | **Source** | **Co.** | **Comments** | **Reference** |
| --- | --- | --- | --- | --- | --- | --- | --- |
| FN557490.1 | L. seeligeri /  str. SLCC3954 | 1/2b | - | Environment (soil isolate) | Germany 1983 | Soil isolate  Non pathogenic | Steinweg et al., J. Bacteriol. 2010 |
| CM001051 | L. seeligeri /  str. FSL N1-067 | - | - | Food | USA | Food processing plant  Hemolytic activity  Non pathogenic | Den Bakker HC et al., BMC Evol Biol 2010 |
| CM001052 | L. seeligeri /  str. FSL S4-171 | 4c | - | Environment | USA 2001 | Urban environment  Non pathogenic | Den Bakker HC et al., BMC Evol Biol 2010 |
| NC_016011.1 | L. ivanovii  subsp. ivanovii /  str. PAM 55 | 5 | - | Animal | Spain 1997 | Isolated from an outbreak of abortion in sheep | Buchrieser et al., J. Bacteriol. 2011 |
| CP007172 | L. ivanovii /  str. WSLC3009 | 5 | - | - | - | Free of intact prophages | Klumpp et al. Genome Announc. 2014 |
| CP009577 | L. ivanovii  subsp. ivanovii /  str. WSLC 3010 | 5 | - | Animal | - | Ruminants | Mario Hupfeld et al., Genome Announc. 2015 |
| CP009576 | L. ivanovii  subsp. londoniensis /  str. WSLC 30151 | 5 | - | Animal | - | Ruminants | Mario Hupfeld et al., Genome Announc. 2015 |
| CM001050 | L. ivanovii/  str. FSL F6-596 | - | - | Food | France | - | Hain T et al., 2006  Den Bakker HC et al., BMC Evol Biol 2010 |
| CP009575 | L. ivanovii subsp. londoniensis /  str. WSLC 30167 | 5 | - | Animal | - | Ruminants | Mario Hupfeld et al., Genome Announc. 2015 |
| AM263198.1 | L. welshimeri /  str. SLCC5334 | 6b | - | Plant | USA 1968 | Decaying plants  Non pathogenic | Hain et al., J. Bact. 2006 |
| CM001049 | L. innocua /  str. FSL J1-023 | - | - | Unknown | - | Hemolytic activity  Non pathogenic  Obtained from Qualicon, exact origin unknown | Den Bakker HC et al., BMC Evol Biol 2010 |
| CM001048 | L. innocua /  str. FSL S4-378 | - | - | Environment | USA 2002 | Non-hemolytic  Puddle of water  Non pathogenic | Den Bakker HC et al., BMC Evol Biol 2010 |
| NC_003212.1 | L. innocua /  str. Clip11262 | 6a | - | Food (cheese) | Morocco 1988 | Non pathogenic | Glaser et al., Science 2001 |
| CP013722 | L. mono. /  str. Lm 3163 | 1/2a | II | Human (blood) | Switzerland 2005 | Tomme cheese listeriosis outbreak | Tasara et al., Genome Annouc. 2016 |
| NC_003210.1 | L. mono. /  str. EGD-e | 1/2a | II | Animal (guinea pig) | UK 1926 | Efficient invasion into epithelial cells | Glaser et al., Science 2001 |
| NC_018588.1 | L. mono. /  str. SLCC2372 | 1/2c | II | Human | UK 1935 | Listeriosis outbreak | Haase et al., Env. Microbiology 2011 |
| NC_017546.1 | L. mono. /  str. FSL R2-561 | 1/2c | II | Food | - 2010 | Listeriosis food-born pathogen | Acccession :PRJNA17317  unpublished |
| NC_018589.1 | L. mono. /  str. SLCC2479 | 3c | II | Food | - 1966 | Attenuation of epithelial cell invasion ability | Haase et al., Env. Microbiology 2011  Kuenne et al. BMC Genomics 2013 |
| HG813249.1 | L. mono. /  str. 6179 | 1/2a | II | Food  (cheese) | Ireland 2014 | Persistent strain  Farmhouse cheese | Casey et al., Front. Microbiol. 2014 |
| NC_020557.1 | L. mono. /  str. La111 | 1/2a | II | Food (cold-smoked salmon) | Denmark 1996 | Persistent strain, lower virulence than clinical ones | Holch et al., Appl. Env. Microb. |
| NC_020558.1 | L. mono. /  str. N53-1 | 1/2a | II | Environment  (smokehouse) | Denmark 2002 | Persistent strain, lower virulence than clinical ones | Holch et al., Appl. Env. Microb. |
| NC_017545.1 | L. mono. /  str. J0161 | 1/2a | II | Human | USA 2000 | Listeriosis outbreak caused by sliced turkey | Olsen et al., Clin. Infect. Dis. 2005 |
| CP007689 | L. mono. /  str. L2074 | 1/2a | II | Human | USA 2010 | Celeri associated outbreak | Gaul LK et al., Clin Infect Dis, 2012 |
| NC_021837.1 | L. mono. /  str. J2-031 | 1/2a | II | Animal  (cow) | USA 1996 | - | Zhang W et al., J Food Prot 2004, Lomonaco S. et al., Appl. Environ.Microbiol. 2011 |
| NC_018593.1 | L. mono. /  str. SLCC7179 | 3a | II | Food (cheese) | Austria 1986 | Attenuation of epithelial cell invasion ability | Haase et al., Env. Microbiology 2011  Kuenne et al. BMC Genomics 2013 |
| CP013724 | L. mono. /  str. Lm N1546 | 1/2a | II | Human  (blood) | Switzerland  2005 | Tomme cheese listeriosis outbreak | Tasara et al., Genome Annouc. 2016 |
| HG813247.1 | L. mono. /  str. R479a | 1/2a | II | Food  (smoked salmon) | Denmark 1996-1999 | Contains typical Listeria pathogenicity island and a full-length internalin AB locus (inlAB like in EGD-e except for homologs of lmo0320 (vip) and lmo2026 | Institute for Milk Hygiene, University of Veterinary Medicine Vienna, 2014 |
| CP009258.1 | L. mono. /  str. Lm60 | 1/2a | II | Human | Switzerland 2014 | Fast cold-adapting strain, isolated from human listeriosis | Tasara et al. Genome Announcement 2014 |
| NC_013766.1 | L. mono. /  str. 08-5578 | 1/2a | II | Human | Canada 2008 | Listeriosis caused by ready-to-eat meat products | Gilmour et al., BMC Genomics 2013 |
| NC_013768.1 | L. mono. /  str. 08-5923 | 1/2a | II | Human | Canada 2008 | Listeriosis caused by ready-to-eat meat products | Gilmour et al., BMC Genomics 2013 |
| CP007687 | L. mono. /  str. L2625 | 1/2a | II | Human | USA 2011 | Cantaloupe associated outbreak | McCollum JT et al., N Engl J Med, 2014 |
| CP011397 | L. mono. /  str. CFSAN007956 | - | II | - | USA | - | Center for Food Safety and Applied Nutrition, United States (FDA) |
|  |  |  |  |  |  |  |  |
| NC_017547.1 | L. mono. /  str. Finland 1998 | 3a | II | Food (dairy butter) | Finland 1998 | Listeriosis outbreak causing 25 cases | Lyytikainen et al., J. Infect. Dis. |
| NC_021823.1 | L.mono. /  str. C1-387 | 1/2a | II | Food (turkey breast) | USA 1999 | - | Den Bakker H et al., BMC Evol Biol 2008 |
| CM003382 | L. mono. /  str. JF5171 | 1/2a;3a | II | Animal  (bovine) | - | Isolated from a bovine placenta in the context of abortion | Rupp S. et al., Vet. Microbiol. 2015 |
| CP013723 | L. mono. /  str. Lm 3136 | 1/2a | II | Human  (blood) | Switzerland  2005 | Tomme cheese listeriosis outbreak | Tasara et al., Genome Annouc. 2016 |
| NC_018592.1 | L. mono. /  str. SLCC5850 | 1/2a | II | Animal (rabbit) | UK 1924 | Slow growth | Haase et al., Env. Microbiology 2011  Kuenne et al. BMC Genomics 2013 |
| CP007160.1 | L. mono. /  str. WSLC1001 | 1/2a | II | Human | Scotland 1935 | Human, spinal fluid | Klumpp et al., Genome Announc. 2014 |
| NC_022568.1 | L. mono. /  str. EGD | 1/2a | II | Animal (guinea pig) | UK 1926 | - | Becavin et al., MBio 2014 |
| CP007684 | L. mono. /  str. L2626 | 1/2a | II | Human | USA | Cantaloupe associated outbreak | McCollum JT et al., N Engl J Med, 2014 |
| CP007688 | L. mono. /  str. L1846 | 1/2b | II | Human | USA 2010 | Hog head cheese associated outbreak | CDC, 2011 |
| CP007685 | L. mono. /  str. L2676 | 1/2a | II | Human | USA 2011 | Cantaloupe associated outbreak | McCollum JT et al., N Engl J Med, 2014 |
| NC_017544.1 | L. mono. /  str. 10403S | 1/2a | II | Human  (skin lesions) | USA 1964 | Isolated from an outbreak of abortion in sheep | Becavin et al., MBio 2014 |
| NC_017728.1 | L. mono. /  str. 07PF0776 | 4b | I | Human (blood culture) | USA 2011 | Fatal myocardial abscess | Mc Mullen et al., J. Bacteriol. 2012 |
| NC_018642.1 | L.mono./  str. L312 | 4b | I | Food (cheese) | - | Efficient invasion into epithelial cells | Chatterjee et al., Int. J. Med. Microb. 2006 |
| CP011004 | L. mono. /  str. N2306 | 4b | I | Human  (blood) | Switzerland 2013-2014 | Listeriosis outbreak caused by ready-to-eat salads | Tasara et al., Genome Announc. 2015 (Pubmed:26021930) |
| FM242711.1 | L. mono. /  str. Clip80459 | 4b | I | Food (rillettes, paté-like meat product) | France 1999 | Listeriosis outbreak | Hain et al., BMC Genomics 2012 (Pubmed:22530965) |
| CP011398 | L. mono. /  str. CFSAN008100 | - | I | Food | - | - | Center for Food Safety and Applied Nutrition, United States (FDA) |
| CP007686 | L. mono. /  str. L2624 | 1/2b | I | Human | USA 2011 | Cantaloupe associated outbreak | McCollum JT et al., N Engl J Med, 2014 |
| NC_021824.1 | L. mono. /  str. J2-064 | 1/2b | I | Animal  (cow) | USA 1989 | Listeriosis outbreak | Gray et al., Appl Environ Microbiol, 2004 |
| NC_018586.1 | L. mono. /  str. SLCC2540 | 3b | I | Human | USA 1956 | Listeriosis outbreak | Haase et al., Env. Microbiology 2011  Kuenne et al. BMC Genomics 2013) |
| NC_018587.1 | L. mono. /  str. SLCC2755 | 1/2b | I | Animal (chinchilla) | USA 1967 | Efficient invasion into epithelial cells | Haase et al., Env. Microbiology 2011) |
| NC_021826.1 | L. mono. /  str. N1-011A | 1/2b | I | Environment | USA | - | Accession:  PRJNA203445 |
| NC_018591.1 | L. mono. /  str. SLCC2482 | 7 | I | Human | USA 1966 | Attenuation of epithelial cell invasion ability | Haase et al., Env. Microbiology 2011  Kuenne et al. BMC Genomics 2013 |
| NC_021838.1 | L. mono. /  str. R2-502 | 1/2b | I | Food (chocolate milk) | USA 1994 | Listeriosis caused by ready-to-eat meat products | Arguedas-Villa C. et al., International Journal of Food Microbiology 2010 |
| CM001159 | L. mono. /  str. ScottA | 4b | I | Human | USA 1983 | - | Yves Briers et al., J. B. 2011  He W., Luchansky J. B. 1997 |
| NC_021830.1 | L. mono. /  str. J1-220 | 4b | I | Human | USA 2002 | Listeriosis outbreak associated with vegetables | Chen et al., J. Bacteriol. 2011 |
| CP013289 | L. mono. /  str. WSLC 1047/  str. SLCC 1090 | 4b | I | Human (cerebrospinal fluid) | Germany 1960 | Female newborn with listeriosis which caused its death | Sumrall E. et al., Genome Annouc. 2016 |
| NC_018584.1 | L. mono. /  str. ATCC 19117 | 4d | I | Animal  (sheep) | USA | Attenuation of epithelial cell invasion ability | Haase et al., Env. Microbiology 2011  Dongyou Liu et al.,J Med Microbiol 2003  Chartterjee SS et al, Int J Med Microbiol 2006  Kuenne et al. BMC Genomics 2013 |
| CP012021 | L. mono. /  str. CFSAN023463 | - | I | - | USA | - | Center for Food Safety and Applied Nutrition, United States (FDA) |
| CP007600.1 | L. mono. /  str. CFSAN006122 | - | I | Food (cheese) | USA 2013 | Listeriosis outbreak from food | Accession:  PRJNA215355 |
| CP010346 | L. mono. /  str. IZSAM_Lm_hs2008 | 4b | I | Human | Italy 2009 | Patient showed typical symptoms of fever, altered mental status, rigor nuchalis, and lethal exitus (isolated from cerebro-spinal fuild) | Patrizia Centorame et al., Genome Annouc. 2015 |
| NC_021840.1 | L. mono. /  str. J1926 | 4b | I | Human (turkey deli) | USA 2002 | Listeriosis outbreak | Accession:PRJNA203445  Y. Chen et al.,J Clin Microbiol. 2008 |
| NC_021827.1 | L. mono. /  str. J1817 | 4b | I | Environment  (turkey deli) | USA 2002 | Listeriosis outbreak | Accession:  PRJNA203445,  Y. Chen et al.,J Clin Microbiol. 2007 |
| NC_021829.1 | L.mono./  str. J1816 | 4b | I | Human | USA 2002 | Listeriosis outbreak caused by turkey deli meat | Chen et al., J. Bacteriol. 2011 |
| CP007492.1 | L. mono. /  str. NE dc2014 | - | I | Food  (cheese) | USA 2014 | Listeriosis outbreak from food | Accession: PRJNA241322 |
| CP009897 | L. mono. /  str. NTSN | 4b | I | Animal | China 2013 | Highly pathogenic brain invasive strain (Sheep) | JIAO Xin-An et al., Chinese Journal of Zoonoses 2013  Tan et al., Genome Announc. 2015 |
| NC_002973.6 | L.mono./  str. 4b str F2365 | 4b | I | Food  (Jalisco Soft Cheese) | USA 1985 | Listeriosis outbreak, mostly pregnant women | Nelson et al., N.A.R. 2004 |
| CP013285 | L. mono./  str. WSLC 1018/  str. ATCC 19118 | 4e | I | - | - | - | Sumrall E. et al., Genome Annouc. 2016 |
| NC_018585.1 | L.mono/  str. SLCC2378 | 4e | I | Animal (poultry) | - | Attenuation of epithelial cell invasion ability | Haase et al., Env. Microbiology 2011  Kuenne et al. BMC Genomics 2013 |
| CP007210.1 | L. mono./  str. WSLC1042 | 4b | I | Human | Germany | Listeriosis outbreak, free of intact prophages | Klumpp et al., Genome Announc. 2014 |
| NC_021825.1 | L.mono./  str. J2-1091 | 1/2a | II | Animal  (cow) | USA 1995 | - | Accession:  PRJNA20344Z |
| NC_021825.1 | L.mono./  J2-1091 | 1/2a | II | Animal  (cow) | USA 1995 | - | Accession:  PRJNA20344Z |
| CM001046 | L. mono/  FSL F2-208 | 4a | III(C) | Human | USA 1999 | Human blood | Den Bakker HC et al., BMC Evol Biol 2010 |
| CP013287 | L. mono. /  str. WSLC 1020 /  str. ATCC 19114 | 4a | III | Animal  (tissue) | England 1931 | - | Sumrall E. et al., Genome Annouc. 2016 |
| NC_018590.1 | L. mono. /  str. SLCC2376 | 4c | III | Animal (poultry) | USA | Attenuation of epithelial cell invasion ability  Poultry | Haase et al., Env. Microbiology 2011  Kuenne et al. BMC Genomics 2013 |
| CP009242 | L. mono /  str. LM850658 | 4a | III | Animal  (sheep) | Australia  1985 | Diseased sheep outbreak | Chun Fang, J. Microbiol. Biotechnol.,  2016 |
| NC_017529.1 | L. mono. /  str. L99 | 4a | III(A) | Food (cheese) | Netherlands 1950 | Non pathogenic | Hain et al., BMC Genomics 2012 |
| NC_017537.1 | L. mono /  str. M7 | 4a | III(A) | Food (cow's milk) | China 2007 | Non pathogenic  Pasteurized cow’s milk | Chen et al., J. Bacteriol. 2011 |
| CM001469 | L. mono. /  str. FSL J1-208 | 4a | IV | Animal (goat) | USA 1998 | Listeriosis outbreak | Kathrin Rychli et al. Plos One, 2014  Den Bakker HC et al., BMC Evol Biol 2012 |
