# Supplementary material for: Unraveling the evolution and coevolution of small regulatory RNAs and coding genes in Listeria
Source: BMC Genomics. 2017 Nov 16;18:882. doi: 10.1186/s12864-017-4242-0 (PMC5689173; doi:10.1186/s12864-017-4242-0)
Supplement: Supplementary file 3 — Ancestral presence/absence patterns of L.m. EGD-e regulatory sRNAs. For all 52 L.m. EGD-e variable sRNAs, the table includes the following information according to the Listeria reference tree (see Fig. 2): root presence - absence information (Root state column), tree branches labels where gain (Gains column) and loss events (Losses column) were inferred (labels correspond to branch identifiers indicated in the cladogram of Fig. 2). The undefined column corresponds to tree branch labels with undefined state due to missing data in the corresponding genomes (draft genomes). The pattern_type column corresponds to the three different types of phyletic profiles inferred: monophyletic, polyphyletic or paraphyletic profiles. (DOCX 128 kb) [file 12864_2017_4242_MOESM3_ESM.docx]

### **Table S3 -** **Ancestral presence/absence patterns of L.m. EGD-e regulatory sRNAs**

| **sRNA** | **Root state** | **Gains** | **Losses** | **Undefined** | **Pattern_type** |
| --- | --- | --- | --- | --- | --- |
| rli107 | present | - | 135,139,140 | - | paraphyletic |
| rli109 | present | - | 154 | - | paraphyletic |
| rli110 | present | - | 122 | - | paraphyletic |
| rli115 | absent | 21,62,74,122,126 | 36 | 135,136,137,138 | paraphyletic |
| rli116 | absent | 7,35,63,69,100,104,126,138,143,156 | 14 | 153,154,155 | paraphyletic |
| rli117 | absent | 3 | 6,9,35,55,63,69,122,133 | - | paraphyletic |
| rli118 | present | - | 142 | 8,9,14,17,153,154,155 | paraphyletic |
| rli122_rli112_rli78_rli50_rli28 | present | - | 8,18,76,95,102,113,117,119,133,139,145,147,156 | 153,154,155 | paraphyletic |
| rli123 | present | - | 5,135,139,140 | - | paraphyletic |
| rli125_rli8-rliC_rli85 | absent | 18,126 | 46,62,63 | - | paraphyletic |
| rli126 | present | 63,69 | 18,95,117,120,133,139,142,152 | 143,144,145,153,154,155 | paraphyletic |
| rli131 | present | - | 154 | - | paraphyletic |
| rli132 | absent | 75 | 96,103,119,133 | 143,144,145 | paraphyletic |
| rli133 | absent | 3 | 6 | 8,9,14,17,134,135,136,137,138,152,153,154,155,156 | paraphyletic |
| rli135 | present | - | 147 | - | paraphyletic |
| rli137 | present | - | 155 | - | paraphyletic |
| rli141 | present | 10,17,26,53,155 | 4,61,62,81,92,104,117,120,133,140 | 50,51,52,56,57,58,94,95,96,135,136,137,138 | paraphyletic |
| rli142 | present | - | 139 | - | paraphyletic |
| rli146 | absent | 3 | - | 134,135,136,137,138,143,144,145,153,154,155 | monophyletic |
| rli16-rliF_rli95 | present | - | 6,8,139,145,147,152 | 153,154,155 | paraphyletic |
| rli22 | present | - | 145 | - | paraphyletic |
| rli24 | present | - | 6 | 8,9,14,17,135,136,137 | paraphyletic |
| rli26 | present | - | 6,136,140 | 8,9,14,17 | paraphyletic |
| rli27 | present | - | 139,147,152 | - | paraphyletic |
| rli28-2_rli45 | present | - | 152 | - | paraphyletic |
| rli28-3 | present | - | 17,134,139,152 | - | paraphyletic |
| rli30 | present | - | 6,17,137,139,142,152 | - | paraphyletic |
| rli33-3_rli33 | absent | 3 | 5 | - | paraphyletic |
| rli34 | absent | 3,143 | 7 | 8,9,14,17,135,136,137 | paraphyletic |
| rli34-2 | present | 94,101,113,117,126 | 14,18,75,140 | 135,136,137,138,152,153,154,155,156 | paraphyletic |
| rli35_rli25_rli23 | present | - | 14,18,136,154,156 | - | paraphyletic |
| rli36 | present | - | 154 | - | paraphyletic |
| rli38 | absent | 132 | - | - | monophyletic |
| rli48 | absent | 10,50,54,82,87,90,134,146 | 58,61,96,97,111,117,120,124 | 135,136,137,138 | paraphyletic |
| rli49 | absent | 3 | 5 | 8,9,14,17,134,135,136,137,138,143,144,145 | paraphyletic |
| rli5-rliA_rli121 | absent | 92,120,126 | 97 | 8,9,14,17 | paraphyletic |
| rli51 | present | - | 139 | 135,136,137,152,153,154,155,156 | paraphyletic |
| rli55 | present | - | 138 | 8,9,14,17,143,144,145,153,154,155 | paraphyletic |
| rli57 | present | - | 122,137 | - | paraphyletic |
| rli6-rliB | absent | 7,75 | 123 | 8,9,14,17,134,135,136,137,138,143,144,145,153,154,155 | paraphyletic |
| rli62 | absent | 10,57,67,82,111,121,132,146 | - | 8,9,14,17,153,154,155 | polyphyletic |
| rli72 | present | - | 48 | - | paraphyletic |
| rli74 | absent | 3 | - | - | monophyletic |
| rli75 | present | - | 5,76,95,139,140 | - | paraphyletic |
| rli76 | present | - | 147 | - | paraphyletic |
| rli79 | present | - | 134,14 | - | paraphyletic |
| rli82 | present | - | 139,141 | - | paraphyletic |
| rli84 | present | - | 17,154 | 135,136,137 | paraphyletic |
| rli9-rliH | present | - | 137 | - | paraphyletic |
| rli94_rli45 | present | - | 154 | - | paraphyletic |
| rli97 | present | - | 137 | - | paraphyletic |
| rli99_rli140 | absent | 17,50,95,104,125,126 | - | 143,144,145,153,154,155 | polyphyletic |

*For all 52 L.m. EGD-e variable sRNAs, the table includes the following information according to the Listeria reference tree (see figure 2): root presence - absence information (Root state column), tree branches labels where gain (Gains column) and loss events (Losses column) were inferred (labels correspond to branch identifiers indicated in the cladogram of figure 2). The undefined column corresponds to tree branch labels with undefined state due to missing data in the corresponding genomes (draft genomes). The pattern_type column corresponds to the three different types of phyletic profiles inferred: monophyletic, polyphyletic or paraphyletic profiles*.
