# Supplementary material for: Unraveling the evolution and coevolution of small regulatory RNAs and coding genes in Listeria
Source: BMC Genomics. 2017 Nov 16;18:882. doi: 10.1186/s12864-017-4242-0 (PMC5689173; doi:10.1186/s12864-017-4242-0)
Supplement: Supplementary file 4 — Listeria sRNAs and coding genes coevolution groups. For each sRNA, the table includes the following informations on the corresponding co-evolving elements: the gene locus tag name (‘Element’ column), the type of element (CDS or 5’UTR, ‘Type’ column), the type of dependency model that highlighted the interaction: x = evolution of the sRNA depends on the state of the 5’UTR/CDS, y = evolution of 5’UTR/CDS depends on the state of the sRNA and xy = bidirectional dependency between evolution of the sRNA and the 5’UTR/CDS element (Model column), the distance between the sRNA and the element in nucleotides (Distance column) and the description of the gene/operon function according to Listeriomics database (‘Description’ column); ‘id’ = identical content. Coevolution groups that are included in the main network hub are highlighted in gray. (DOCX 142 kb) [file 12864_2017_4242_MOESM4_ESM.docx]

**Table S4 - *Listeria* sRNA and coding genes coevolution groups**

| **sRNA** | **Element** | **Type** | **Model** | **Distance** | **Description** |
| --- | --- | --- | --- | --- | --- |
| rli107 | lmo0082 | cds | y | 397659 | Unknown ; Operon 014 (lmo0079, lmo0080, lmo0081, lmo0082) ; Close to Lmo0079 similar to a bacillus transposase |
|  | lmo0203 | cds | y | 519496 | Metalloproteaze precursor (elastase) ;Belongs to Listeria Pathogenicity Island LIPI-1 extended ;Operon 033 (5 genes lm0203, lmo0204, lmo0205, lmo0206, lmo0207) |
|  | lmo0203 | cds | xy | 519496 | id |
|  | lmo0206 | cds | y | 524123 | Distant similarity with viral glycoprotein gp160 of HIV type 1 ;Belongs to Listeria Pathogenicity Island LIPI-1 extended ;Operon 033 (5 genes lm0203, lmo0204, lmo0205, lmo0206, lmo0207) |
|  | lmo0206 | cds | xy | 524123 | id |
| rli115 | lmo0069 | utr | xy | 1766 | homolog within ESAT-6 gene cluster ; Belongs to Operon 012 (lmo0067, lmo0068, lmo0069) |
|  | lmo0069 | utr | y | 1766 | id |
|  | lmo0069 | utr | x | 1766 | id |
|  | lmo0070 | utr | xy | 1336 | Flagellar biosynthesis pathway component FlhB ; (cell motility and secretion, intracellular trafficking and secretion) ; Belongs to Operon 013 (lmo0070, lmo0071, lmo0072, lmo0073, lmo0074) |
|  | lmo0070 | utr | y | 1336 | id |
|  | lmo0070 | utr | x | 1336 | id |
|  | lmo0071 | utr | xy | 449 | hypothetical protein ; Belongs to Operon 013 (lmo0070, lmo0071, lmo0072, lmo0073, lmo0074) |
|  | lmo0071 | utr | y | 449 | id |
|  | lmo0071 | utr | x | 449 | id |
|  | lmo0073 | cds | y | 226 | Belongs to Operon 013 (lmo0070, lmo0071, lmo0072, lmo0073, lmo0074) |
|  | lmo0073 | cds | xy | 226 | id |
|  | lmo0073 | cds | x | 226 | id |
|  | lmo0073 | utr | y | 204 | id |
|  | lmo0073 | utr | x | 204 | id |
|  | lmo0074 | cds | y | 498 | Belongs to Operon 013 (lmo0070, lmo0071, lmo0072, lmo0073, lmo0074) |
|  | lmo0074 | cds | xy | 498 | id |
|  | lmo0074 | cds | x | 498 | id |
| rli116 | lmo0146 | utr | y | 1254 | hypothetical protein |
| rli117 | lmo0082 | cds | y | 59343 | Unknown ; Operon 014 (lmo0079, lmo0080, lmo0081, lmo0082) ; Close to Lmo0079 similar to a bacillus transposase |
|  | lmo0082 | cds | xy | 59343 | id |
|  | lmo0263 | utr | xy | 138467 | internalin H (inlH) ; Bacterial adhesion/invasion protein |
|  | lmo0263 | utr | x | 138467 | id |
|  | lmo0549 | cds | xy | 441170 | Similar to internalin protein |
|  | lmo0549 | cds | x | 441170 | id |
|  | lmo2470 | cds | xy | 544666 | Internalin P (inlP) ; Bacterial adhesion/invasion protein |
|  | lmo2470 | cds | x | 544666 | id |
| rli123 | lmo0082 | cds | y | 776710 | Unknown ; Operon 014 (lmo0079, lmo0080, lmo0081, lmo0082) ; Close to Lmo0079 similar to a bacillus transposase |
|  | lmo0095 | cds | y | 759102 | hypothetical protein |
|  | lmo0095 | cds | x | 759102 | id |
|  | lmo0206 | cds | y | 650249 | Distant similarity with viral glycoprotein gp160 of HIV type 1 ; Belongs to Listeria Pathogenicity Island LIPI-1 extended ; Operon 033 (5 genes lm0203, lmo0204, lmo0205, lmo0206, lmo0207) |
|  | lmo2733 | cds | y | 1000068 | Similar to PTS system fructose specific IIABC component ; Operon 494 (lmo2733, lmo2734, lmo2735, lmo2736) |
| rli125_rli8-rliC_rli85 | lmo1116 | cds | x | 536 | Similar to regulatory proteins ; Arabinose operon control ; Operon 180 (lmo116, lmo1117) |
|  | lmo1116 | utr | x | 1310 | id |
|  | lmo1117 | cds | x | 115 | This domain superfamily is found in a variety of structurally ; related metalloproteins, including the type I extradiol ; dioxygenases, glyoxalase I ; And a group of antibiotic resistance proteins; ; Operon 180 (lmo1116, lmo1117) |
|  | lmo1117 | utr | x | 472 | id |
| rli132 | lmo2026 | utr | y | 1412 | Similar to ferredoxin (energy profuction and conversion) |
|  | lmo2026 | utr | x | 1412 | id |
|  | lmo2027 | utr | y | 18 | Bacterial adhesion/invasion protein, soluble internalin |
|  | lmo2027 | utr | x | 18 | id |
| rli133 | lmo0017 | utr | y | 820307 | Similar to Bacillus anthracis CapA protein (polyglutamate capsule biosynthesis) |
|  | lmo0017 | utr | x | 820307 | id |
|  | lmo0082 | cds | y | 884781 | Unknown ; Operon 014 (lmo0079, lmo0080, lmo0081, lmo0082) ; Close to Lmo0079 similar to a bacillus transposase |
|  | lmo0082 | cds | xy | 884781 | id |
|  | lmo0206 | cds | y | 1011245 | Distant similarity with viral glycoprotein gp160 of HIV type 1 ; Belongs to Listeria Pathogenicity Island LIPI-1 extended ; Operon 033 (5 genes lm0203, lmo0204, lmo0205, lmo0206, lmo0207) |
|  | lmo0264 | cds | y | 1085098 | Bacterial adhesion/invasion protein, internalin E (inlE) |
|  | lmo0264 | cds | x | 1085098 | id |
|  | lmo0264 | utr | y | 1084999 | id |
|  | lmo0264 | utr | x | 1084999 | id |
|  | lmo0333 | utr | y | 1159716 | Putative pepetidoglycan bound protein (LPXTG motif) ; Internalin-like protein, Internalin I (inlI) |
|  | lmo0333 | utr | x | 1159716 | id |
|  | lmo0334 | cds | x | 1165387 | NADPH-dependent FMN reductase |
|  | lmo0419 | utr | y | 1239491 | hypothetical protein (membrane protein) ; Belongs to Operon 073 (lmo0418, lmo0419) |
|  | lmo0419 | utr | x | 1239491 | id |
|  | lmo0438 | cds | y | 1266398 | Listeria nuclear targeted protein A precursor (lntA) ; Bacterial invasion protein |
|  | lmo0438 | cds | x | 1266398 | id |
|  | lmo0438 | utr | y | 1266299 | id |
|  | lmo0550 | cds | y | 1387945 | cell surface protein, peptidoglycan bound protein |
|  | lmo0550 | cds | x | 1387945 | id |
|  | lmo2107 | utr | y | 41915 | Similar to transcriptional regulator (DeoR family);Transcriptionnal regulators of sugar metabolism;Sugar-phosphate isomerase |
|  | lmo2107 | utr | x | 41915 | id |
|  | lmo2157 | utr | y | 96322 | Alkyl sulfatase and related hydrolases (secondary metabolites biosynthesis,Transport and catabolism) |
|  | lmo2157 | utr | x | 96322 | id |
|  | lmo2470 | cds | y | 398618 | Internalin P (inlP) ; Bacterial adhesion/invasion protein |
|  | lmo2470 | utr | y | 398519 | id |
|  | lmo2470 | utr | x | 398519 | id |
| rli141 | lmo2292 | cds | x | 20979 | 3-oxoacyl-acyl carrier protein synthase involved in fatty acid biosynthesis ; Operon 399 (lmo2200, lmo2199) |
|  | lmo2329 | cds | y | 122 | Similar to putative repressor protein (bacteriophage A118) |
|  | lmo2329 | cds | xy | 122 | id |
|  | lmo2329 | cds | x | 122 | id |
| rli146 | lmo0082 | cds | y | 232556 | Unknown ; Operon 014 (lmo0079, lmo0080, lmo0081, lmo0082) ; Close to Lmo0079 similar to a bacillus transposase |
|  | lmo0082 | cds | xy | 232556 | id |
|  | lmo0203 | cds | y | 109513 | Metalloproteaze precursor (elastase) ; Belongs to Listeria Pathogenicity Island LIPI-1 extended ; Operon 033 (5 genes lm0203, lmo0204, lmo0205, lmo0206, lmo0207) |
| rli26 | lmo0082 | cds | y | 302362 | Unknown ; Operon 014 (lmo0079, lmo0080, lmo0081, lmo0082) ; Close to Lmo0079 similar to a bacillus transposase |
|  | lmo0206 | cds | y | 175901 | Distant similarity with viral glycoprotein gp160 of HIV type 1 ; Belongs to Listeria Pathogenicity Island LIPI-1 extended ; Operon 033 (5 genes lm0203, lmo0204, lmo0205, lmo0206, lmo0207) |
| rli28-3 | lmo0035 | cds | y | 828832 | Similar to PTS system, cellobiose-specific IIC component |
| rli30 | lmo0082 | cds | y | 454442 | Unknown ; Operon 014 (lmo0079, lmo0080, lmo0081, lmo0082) ; Close to Lmo0079 similar to a bacillus transposase |
|  | lmo0183 | cds | y | 352807 | Similar to alpha-glucosidase |
|  | lmo0501 | cds | y | 2423 | Transcriptional antiterminator (BglG family) ; Operon 085 (lmo0501, lmo0502, lmo0502, lmo0503, lmo0504, lmo0505, lmo0506, lmo0507, lmo0508) |
|  | lmo0502 | cds | y | 1817 | Similar to putative sugar-phosphate isomerase ; Operon 085 (lmo0501, lmo0502, lmo0502, lmo0503, lmo0504, lmo0505, lmo0506, lmo0507, lmo0508) |
|  | lmo0503 | cds | y | 1322 | Similar to PTS fructose-specific enzyme IIA component |
|  | lmo0505 | cds | y | 246 | Similar to ribulose-5-phosphate 3-epimerase ; Operon 085 (lmo0501, lmo0502, lmo0502, lmo0503, lmo0504, lmo0505, lmo0506, lmo0507, lmo0508) |
|  | lmo0505 | cds | x | 246 | id |
|  | lmo0508 | cds | y | 1005 | Similar to PTS system (phosphotransferase system), Galactitol-specific IIC component ; Operon 085 (lmo0501, lmo0502, lmo0502, lmo0503, lmo0504, lmo0505, lmo0506, lmo0507, lmo0508) |
|  | lmo0508 | cds | x | 1005 | id |
|  | lmo0551 | cds | y | 48620 | hypothetical protein |
|  | lmo0551 | cds | x | 48620 | id |
|  | lmo2445 | cds | x | 969988 | Similar to secreted internalin |
|  | lmo2732 | cds | y | 679924 | Phosphosugar-binding protein |
| rli33-3_rli33 | lmo0072 | cds | y | 630260 | Uncharacterized protein |
|  | lmo0082 | cds | y | 622098 | Unknown ; Operon 014 (lmo0079, lmo0080, lmo0081, lmo0082) ; Close to Lmo0079 similar to a bacillus transposase |
|  | lmo0082 | cds | xy | 622098 | id |
|  | lmo1001 | cds | y | 323472 | Similar to protein YkvS, uncharacterized protein conserved in bacteria |
| rli34 | lmo0082 | cds | y | 716720 | Unknown ; Operon 014 (lmo0079, lmo0080, lmo0081, lmo0082) ; Close to Lmo0079 similar to a bacillus transposase |
|  | lmo1001 | cds | y | 229301 | Similar to protein YkvS, uncharacterized protein conserved in bacteria |
|  | lmo1001 | cds | xy | 229301 | id |
| rli34-2 | lmo0638 | cds | y | 190 | hypothetical protein |
|  | lmo0638 | cds | xy | 190 | id |
|  | lmo0638 | utr | y | 91 | id |
| rli48 | lmo2272 | cds | y | 176 | hypothetical protein |
|  | lmo2272 | cds | xy | 176 | id |
|  | lmo2272 | cds | x | 176 | id |
|  | lmo2272 | utr | y | 77 | id |
|  | lmo2272 | utr | x | 77 | id |
| rli49 | lmo0072 | cds | y | 362026 | Uncharacterized protein ; Operon 013 (lmo0070, lmo0071, lmo0072, lmo0073) |
|  | lmo0082 | cds | y | 370065 | Unknown ; Operon 014 (lmo0079, lmo0080, lmo0081, lmo0082) ; Close to Lmo0079 similar to a bacillus transposase |
|  | lmo0082 | cds | xy | 370065 | Unknown ; Operon 014 (lmo0079, lmo0080, lmo0081, lmo0082) ; Close to Lmo0079 similar to a bacillus transposase |
| rli5-rliA_rli121 | lmo2309 | cds | y | 1050799 | Hypothetical protein |
|  | lmo2309 | cds | xy | 1050799 | id |
|  | lmo2309 | cds | x | 1050799 | id |
|  | lmo2407 | utr | xy | 960239 | Hypothetical protein |
| rli74 | lmo0082 | cds | y | 123093 | Unknown ; Operon 014 (lmo0079, lmo0080, lmo0081, lmo0082) ; Close to Lmo0079 similar to a bacillus transposase |
|  | lmo0082 | cds | xy | 123093 | id |
|  | lmo0203 | cds | y | 50 | Metalloproteaze precursor (elastase) ; Belongs to Listeria Pathogenicity Island LIPI-1 extended ; Operon 033 (5 genes lm0203, lmo0204, lmo0205, lmo0206, lmo0207) |
| rli75 | lmo0294 | cds | x | 867 | Similar to transcription regulator LysR-gltR family |
|  | lmo0294 | utr | x | 767 | id |
|  | lmo0295 | cds | x | 11 | Similar to FMN-containing NADPH-linked nitro/flavin reductase |
|  | lmo0295 | utr | x | 749 | id |
| rli79 | lmo0082 | cds | y | 610590 | Unknown ; Operon 014 (lmo0079, lmo0080, lmo0081, lmo0082) ; Close to Lmo0079 similar to a bacillus transposase |
|  | lmo0082 | cds | xy | 610590 | id |
|  | lmo0203 | cds | y | 487547 | Metalloproteaze precursor (elastase) ; Belongs to Listeria Pathogenicity Island LIPI-1 extended ; Operon 033 (5 genes lm0203, lmo0204, lmo0205, lmo0206, lmo0207) |
|  | lmo2790 | cds | y | 763335 | Similar to ribose 5-phosphate epimerase ; Operon 477 (10 genes) |
| rli99_rli140 | lmo2303 | cds | y | 7552 | Similar to protein gp66 (Bacteriophage A118) |
|  | lmo2317 | cds | y | 1436 | Similar to gp49 (Bacteriophage A118) |
|  | lmo2317 | cds | xy | 1436 | id |
|  | lmo2317 | cds | x | 1436 | id |
|  | lmo2319 | cds | y | 239 | Similar to bacteriophage protein |
|  | lmo2319 | cds | xy | 239 | id |
|  | lmo2319 | cds | x | 239 | id |
|  | lmo2320 | cds | y | 48 | hypothetical protein |
|  | lmo2320 | cds | xy | 48 | id |
|  | lmo2320 | cds | x | 48 | id |

*For each sRNA the table includes the following informations on the corresponding co-evolving elements: the gene locus tag name (‘Element’ column), the type of element (CDS or 5’UTR, ‘Type’ column), the type of dependency model that highlighted the interaction: x = evolution of the sRNA depends of state of the 5’UTR/CDS, y = evolution of 5’UTR/CDS depends of state of the sRNA and xy = bidirectional dependency between evolution of the sRNA and the 5’UTR/CDS element (Model column), the distance between the sRNA and the element in nucleotides (Distance column) and the description of the gene/operon function according to Listeriomics database (‘Description’ column); ‘id’=identical content. Coevolution groups that are included in the main network hub are highlighted in gray.*
