# Supplementary figures and images for: Unraveling the evolution and coevolution of small regulatory RNAs and coding genes in Listeria
Source: BMC Genomics. 2017 Nov 16;18:882. doi: 10.1186/s12864-017-4242-0 (PMC5689173; doi:10.1186/s12864-017-4242-0)

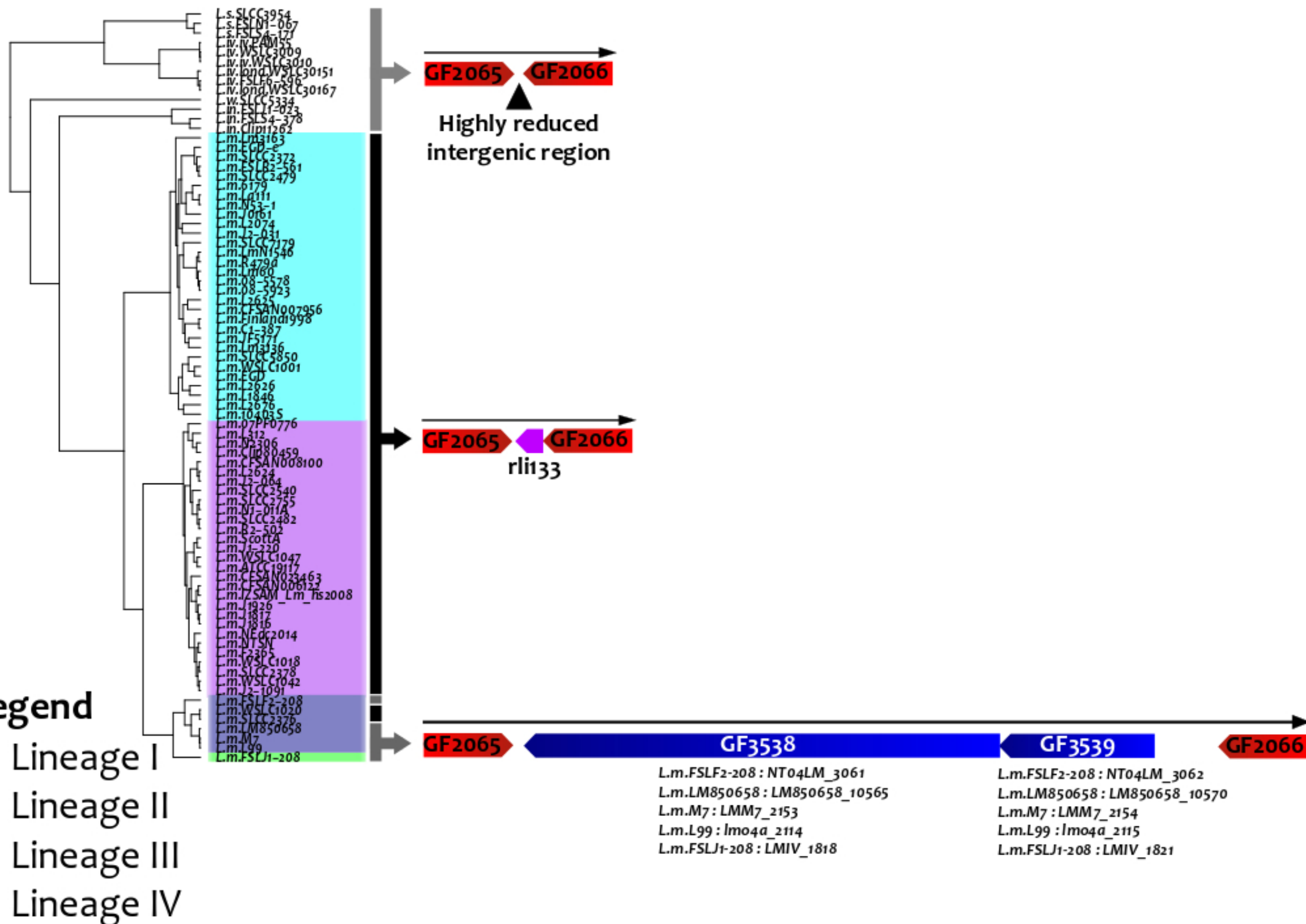

Supplement: Supplementary file 5 — Rli133 genomic context conservation in Listeria. 5′ and 3′ homolog genes are represented using red arrows. GFXXXX names correspond to PanOCT ortholog clusters identifiers. Blue arrows correspond to two genes inserted in several strains of Listeria lineages III and IV. (PDF 212 kb) [file 12864_2017_4242_MOESM5_ESM.pdf]

Distance between sRNAs and genes

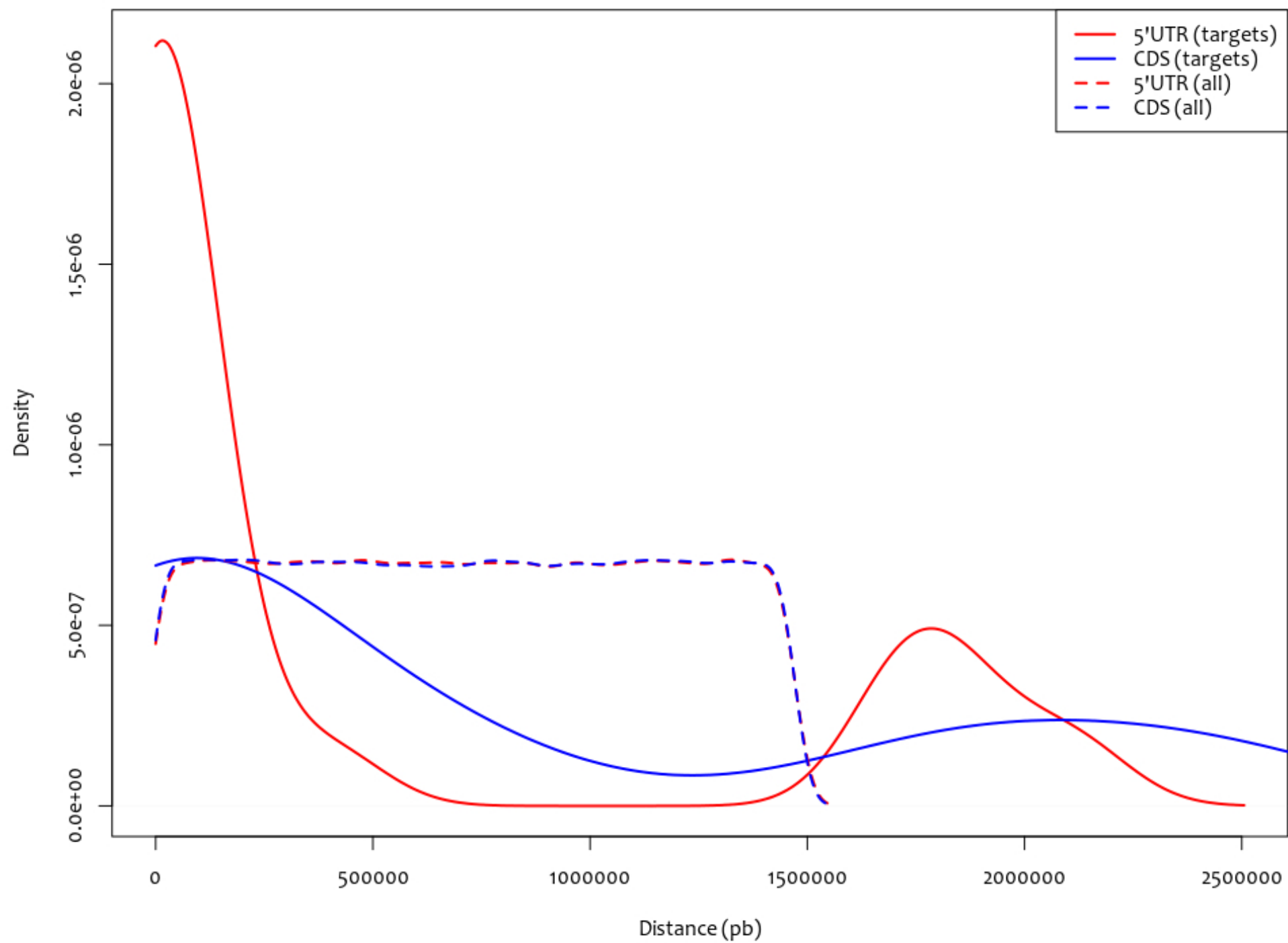

Supplement: Supplementary file 7 — Genomic distance between coevolving sRNAs and CDS. Plain curves show the distance density between sRNAs and 5’UTRs (red) or CDS (blue) engaged in coevolution relationships, considering genome circularity. They are compared to distances between sRNAs and all 5’UTRs or CDS (all) respectively represented by red and blue dotted curves. (PDF 117 kb) [file 12864_2017_4242_MOESM7_ESM.pdf]
