# Supplementary material for: Unraveling the evolution and coevolution of small regulatory RNAs and coding genes in Listeria
Source: BMC Genomics. 2017 Nov 16;18:882. doi: 10.1186/s12864-017-4242-0 (PMC5689173; doi:10.1186/s12864-017-4242-0)
Supplement: Supplementary file 6 — Multiple alignment of rli133. This figure represents the multiple alignment of rli133 sequences in strains where it is present. Red denotes a fully conserved position. The phylogenetic tree at the left corresponds to a Maximum Likelihood tree computed from the corresponding multiple alignment. (PDF 1413 kb) [file 12864_2017_4242_MOESM6_ESM.pdf]

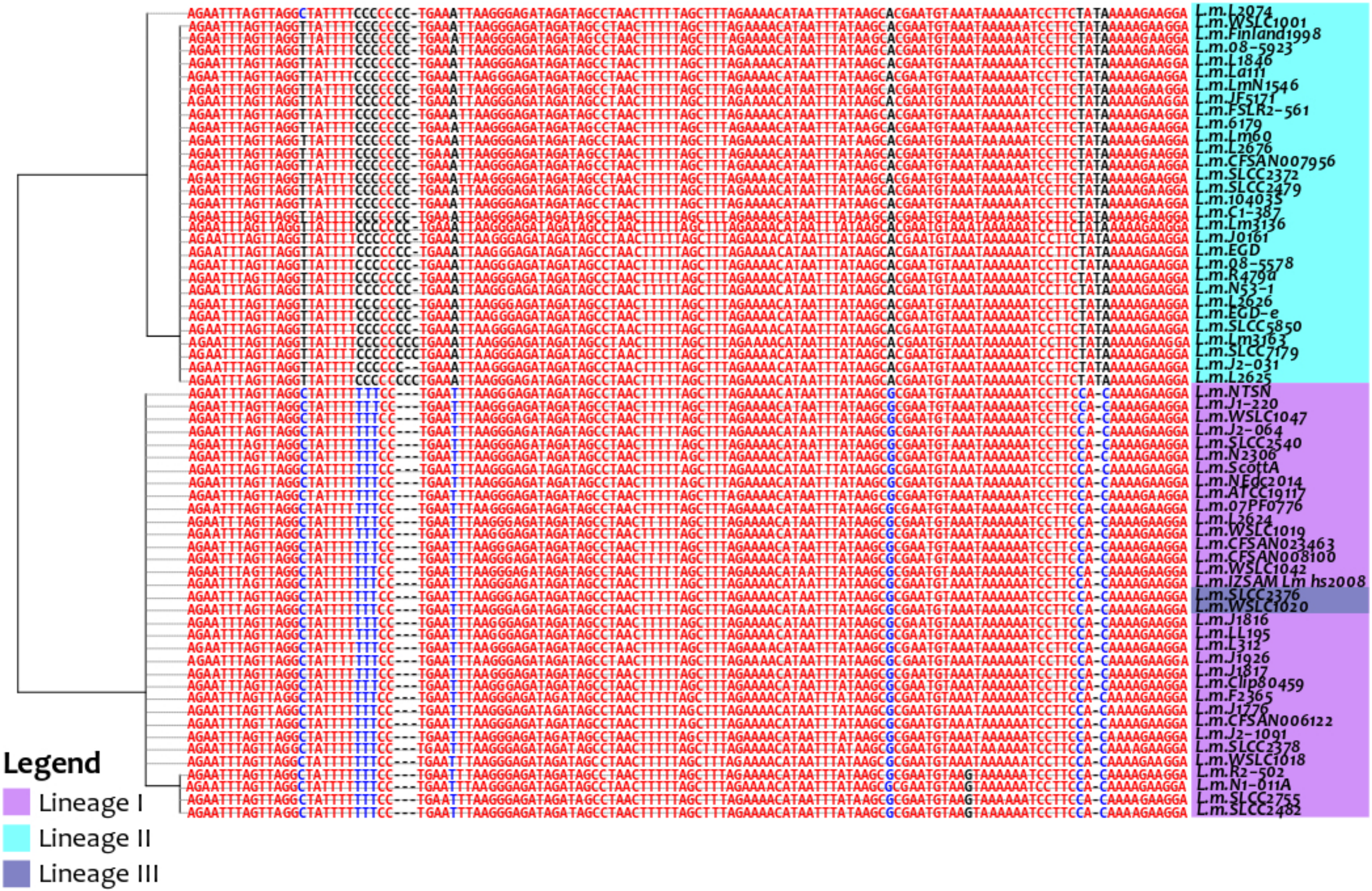
L.m.L2074
L.m.WSLC1001
L.m.Finland1998
L.m.08-5923
L.m.L1846
L.m.La111
L.m.LmN1546
L.m.JF5171
L.m.FSLR2-561
L.m.6179
L.m.Lm60
L.m.L2676
L.m.CFSAN007956
L.m.SLCC2372
L.m.SLCC2479
L.m.104035
L.m.C1-387
L.m.Lm3136
L.m.J0161
L.m.EGD
L.m.08-5578
L.m.R479a
L.m.N53-1
L.m.L2626
L.m.EGD-e
L.m.SLCC5850
L.m.Lm3163
L.m.SLCC7179
L.m.J2-031
L.m.L2625
L.m.NTSN
L.m.J1-220
L.m.WSLC1047
L.m.J2-064
L.m.SLCC2540
L.m.N2306
L.m.ScottA
L.m.NEdc2014
L.m.ATCC19117
L.m.07PF0776
L.m.L2624
L.m.WSLC1019
L.m.CFSAN023463
L.m.CFSAN008100
L.m.WSLC1042
L.m.IZSAM Lm hs2008
L.m.SLCC2376
L.m.WSLC1020
L.m.J1816
L.m.LL195
L.m.L312
L.m.J1926
L.m.J1817
L.m.Clip80459
L.m.F2365
L.m.J1776
L.m.CFSAN006122
L.m.J2-1091
L.m.SLCC2378
L.m.WSLC1018
L.m.R2-502
L.m.N1-011A
L.m.SLCC2755
L.m.SLCC2482
Legend
Lineage I
Lineage II
Lineage III
